# Scope and mechanism of the highly stereoselective metal-mediated domino aldol reactions of enolates with aldehydes

**DOI:** 10.3762/bjoc.12.80

**Published:** 2016-04-27

**Authors:** M Emin Cinar, Bernward Engelen, Martin Panthöfer, Hans-Jörg Deiseroth, Jens Schlirf, Michael Schmittel

**Affiliations:** 1 Department Chemie-Biologie, OC1, Universität Siegen, Adolf-Reichwein-Str., D-57068 Siegen, Germany

**Keywords:** DFT, diastereoselective reaction, domino aldol, metal enolate, tetrahydropyran

## Abstract

A one-pot transformation, which involves the reaction of ketones with aldehydes in the presence of metal halides to furnish tetrahydro-2*H*-pyran-2,4-diols in a highly diastereoselective manner, is investigated thoroughly by experiments and computations. The reaction was also successfully implemented on a flow micro reactor system.

## Introduction

Since its discovery in the late nineteenth century the aldol reaction has become one of the most powerful tools in the field of carbon–carbon bond formation [1–5]. It is widely used in the formation of many natural products [6–11], stereoselective syntheses [12–16], and tandem reactions [17–19]. While the latter processes usually comprise only one aldol reaction, tandem reaction sequences containing two consecutive aldol steps are mostly limited to the trimerization of enolates [20–22].

**Scheme 1:**
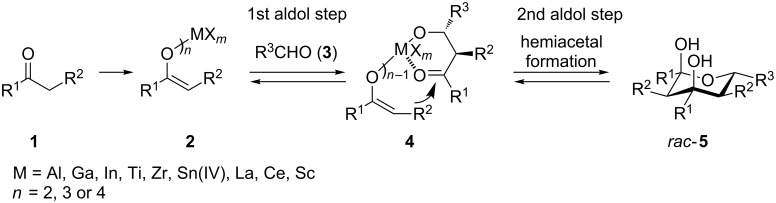
Synthesis of racemic tetrahydro-2*H*-pyran-2,4-diols *rac-***5** from enolates **2** and aldehydes **3**.

Metal enolates (Ti [23], Zr [24], Si [25], and Sn [26]) and boron enolates [27] have adopted a considerable significance because of their high potential to control the stereochemical outcome of the bond formation [28–30]. However, the other group III metal enolates have been almost completely omitted over the years [31]. We have already reported a domino aldol–aldol–hemiacetal process that furnishes racemic tetrahydro-2*H*-pyran-2,4-diols in a highly stereoselective manner (Scheme 1) [30–37].

In this paper, the domino aldol–aldol–hemiacetal reaction involving several metals (Al, Ga, In, Ti, Zr, Sn), and various aldehydes and ketones is studied experimentally and computationally.

## Results

### Metal effect

**Scheme 2:**
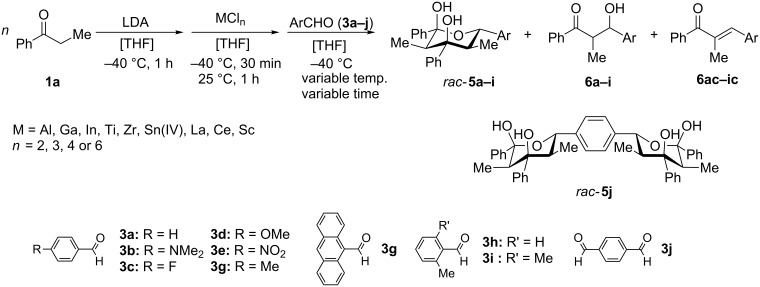
Synthesis of *rac-***5a–j** and monoaldol products **6a–i** and **6ac–ic** as obtained from propiophenone (**1a**) in the presence of metal chloride and substituted arylaldehydes.

The experiments were performed to screen suitable metal fragments for their ability to promote the domino aldol reaction by studying the reaction between propiophenone (**1a**) and benzaldehyde (**3a:** Ar = Ph) (Scheme 2). The enolate was generated from propiophenone by deprotonation with lithium diisopropylamide (LDA) at −40 °C in tetrahydrofuran (THF) and was subsequently reacted with 0.33 equivalents of MCl_3_ or 0.25 equivalents of MCl_4_, respectively. The resulting metal enolate was then treated with a stoichiometric amount of benzaldehyde (**3a**) and stirred for 2 h at 0 °C, room temperature or 67 °C. The hemiacetal **5a** was obtained in varying yields along with some amount of the monoaldol **6a** [38–39] (obtained as a mixture of two diastereomers; *syn/anti* ≈ 1:1) and condensation product **6ac** (Scheme 2, Table 1).

**Table 1:** Effect of metals on the domino aldol reaction of **1a** and **3a** at different temperatures (reaction time: 2 h) on the yield of **5a** and **6a**.

Entry	Metal	Yield of **5a** and **6a** at various temperatures					

		at 0 °C		at 25 °C		at 67 °C	

		**5a** (%)	**6a** (%)	**5a** (%)	**6a** (%)	**5a** (%)	**6a** (%)

1	Al	18	19	64^a^	8	8	−
2	Ga	17 (+20)^b^	22	70	19	67	−
3	In	76	3	85^a^	13	60	2
4	Ti^b^	19	26	38	14	50^a^	5
5	Zr	61	10	76^c^	23	21	−
6	Sn(IV)	7	20	36 (+29)^b,c^	22	17	5

^a^Ref. [34]. ^b^A diastereomer of **5a**. ^c^Ref. [37].

Aside of the ions mentioned in Table 1 the metal-mediated domino aldol reaction was also probed with LaCl_3_, La(OTf)_3_, CeCl_3_, Sc(OTf)_3_, BF_3_ and SnCl_2_ resulting in failure. While SnCl_2_ afforded **6a** in 90% yield, LaCl_3_, La(OTf)_3_, CeCl_3_, and Sc(OTf)_3_ furnished **6a** in 25, 52, 10 and 55% yield, respectively. In almost all cases the mono aldolate **6a** was the main product at lower temperatures, e.g., at 0 °C, along with the domino aldol product **5a** obtained in the range of 7–26% yield.

Higher yields of **5a** were obtained at 0 °C in the presence of In and Zr. Interestingly, in the case of gallium at 0 °C and Sn(IV) at 25 °C a diastereomer of **5a** also formed in 20 and 29% yields, respectively. Generally, with nearly all metals, the yield of **5a** increased dramatically when the temperature was raised to 25 °C, but dropped at higher temperatures. The decreased yield at 67 °C may emerge from the irreversible formation of the aldol condensation product **6ac**, which is obtained in 73% yield in presence of AlCl_3_, on expense of **5a**.

**Figure 1:**
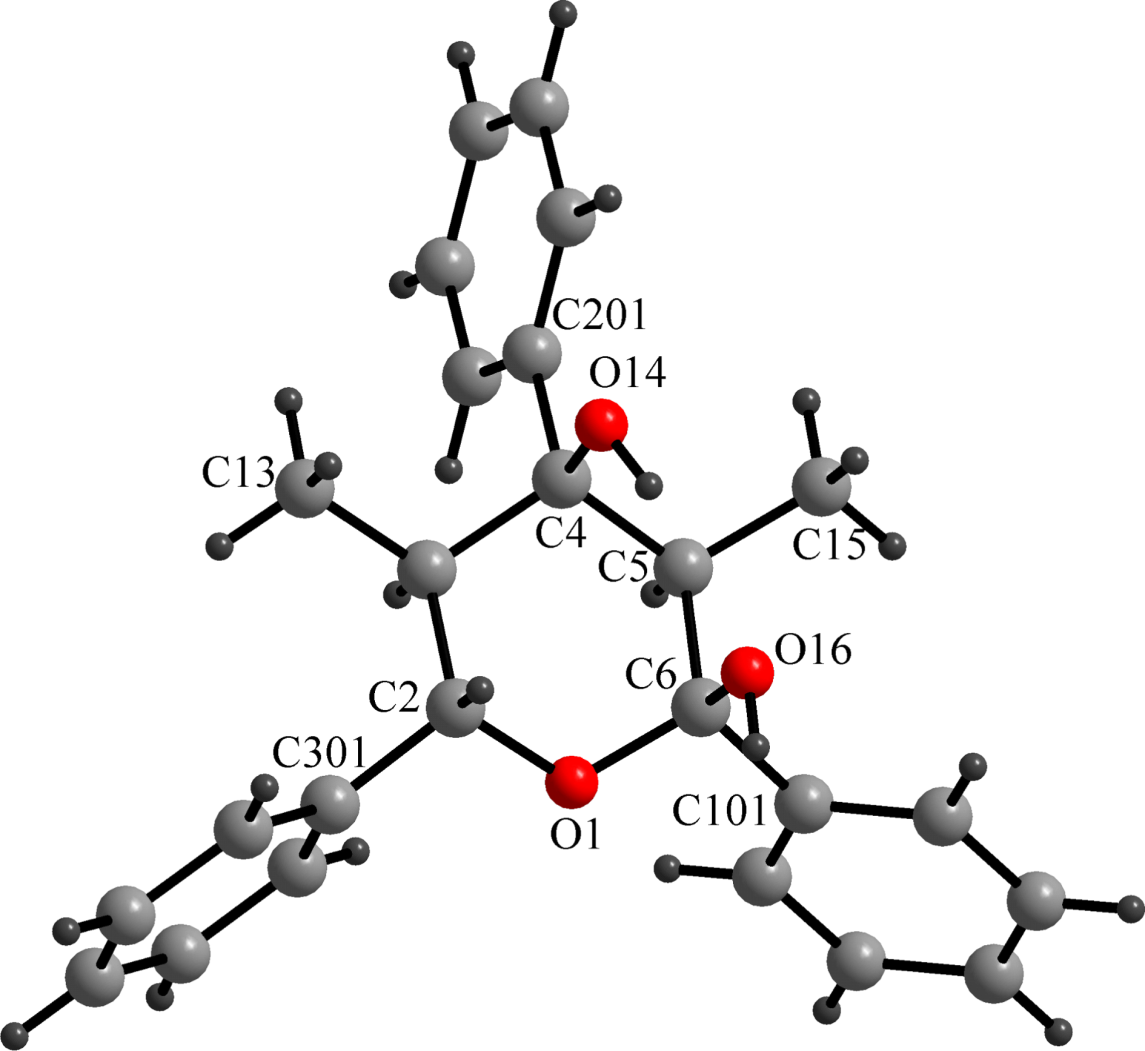
Crystal structure of enantiopure **5a** [40].

The X-ray structure of racemic **5a** (from ethanol) could not be solved due to the presence of a solid racemate in the orthorhombic space group *P*2_1_2_1_2_1_ (no. 19). Three axial hydroxy groups, whose probability of allocation at C2 and C6 is 0.5, respectively, appear to be attached to the pyran ring. This can easily be explained by the superposition of two enantiomers which are statistically and isoconformationally incorporated in the crystal lattice. Separation of two enantiomers was achieved by using chiral column chromatography (Chiralpak AD, Daicel) followed by recrystallization from H_2_O/MeOH (1:4) providing the appropriate crystal for X-ray analysis. Accordingly, the enantiopure crystal of **5a** was unambiguously assigned to a tetrahydro-2*H*-pyran-2,4-diol structure with all phenyl and methyl groups occupying equatorial positions while the hydroxy groups are placed in axial positions with an allocation probability of 1.0 each (Figure 1). Within the crystal lattice the molecules arrange in a chain along the *a*-axis, so that each molecule is twisted by 180° against each other. Additionally, there are alternating inter- and intramolecular hydrogen bonds between the hydroxy groups.

### Mechanistic aspects

To shed light on the mechanism, the metal to enolate ratio was varied (Table 2), while keeping the optimum temperature for each metal as determined in the previous experiments. The reaction was already successful with two equivalents of enolate per metal fragment. However, higher yields were obtained at higher loadings. For example, zirconium worked best with three enolate units and tin with four. Surprisingly, an excess of an enolate had different effects on the reactions depending on the metals. While the yield was decreased with zirconium, it increased both with aluminum and indium. In the latter case, >99% yield was obtained.

**Table 2:** Dependence on the stoichiometric amount of propiophenone (**1a**) enolate with regard to the metal (reaction time: 2 h, reaction temperature: 25 °C, **5a**/**6a** in %).

Entry	Metal chloride	Amount of propiophenone (**1a**)							

		2 equiv		3 equiv		4 equiv		6 equiv	

		**5a**	**6a**	**5a**	**6a**	**5a**	**6a**	**5a**	**6a**

1	ZrCl_4_	33	48	76	23	68	22	–	–
2	SnCl_4_	20	45	14	35	36	22	–	–
3	AlCl_3_	–	–	66	8	–	–	66	34
4	InCl_3_	–	–	85	13	–	–	>99	–

In the same manner the influence of the amount of aldehyde was examined. As can be seen from the data in Table 3 the ratio of **5a** to **6a** decreases dramatically by increasing the amount of aldehyde two-fold, owing to the formation of higher amounts of the monoaldol product in presence of excess aldehyde (Scheme 2).

**Table 3:** Stoichiometry dependence on the amount of aldehyde (reaction time: 2 h, reaction temperature: 25 °C, **5a**/**6a** in %).

Entry	Metal	In presence of benzaldehyde (**3a**) in							

		1 equiv		1.5 equiv		2 equiv		4 equiv	

		**5a**	**6a**	**5a**	**6a**	**5a**	**6a**	**5a**	**6a**

1	Zr	65	7.0	40	16	19	31	5	60
2	Al	66	8	42	39	30	40	–	–
3	In	78	13	53	30	34	42	–	–

In order to check whether this outcome is the result of thermodynamic control, a second aldehyde was added to the reaction mixture after 2 h. The larger the amount of aldehyde in the reaction, the higher is the yield of monoaldol product **6a**, which supports a thermodynamically controlled equilibrium as further confirmed by the following observations: (1) with gallium(III) two diastereomeric tetrahydro-2*H*-pyran-2,4-diols are formed at 0 °C and only one (i.e., **5a**) at elevated temperature; (2) at higher temperature, the product yield of **5a** is gradually reduced on account of new **6ac** (only in the case of AlCl_3_), which is expected to be formed irreversibly from the metal-bound monoaldolate; (3) when excess benzaldehyde (**3a**) was added to the reaction with AlCl_3_, the yield of domino product **5a** gradually decreased from, for example, 42% to 30% with 1.5 and 2.0 equivalents of benzaldehyde, respectively.

Likewise, the amount of metal chloride influences the yield of **5a**. The higher the amount of metal chloride the less likely is the molecular preorganization, which is necessary for the reaction. The reactions carried out with 1.0, 0.75 and 0.5 equivalents of zirconium provided **5a** in 76, 42 and 14% yield, respectively. Reducing the amount to 0.25 equivalents of zirconium furnished only 3% yield.

Even the concentration influences the yield of **5a**. The optimum concentration is 375 mM, in which the reaction afforded 76% yield of **5a**. In case of 750 mM, the yield decreases drastically to 40%. However, lower concentrations such as 250 mM and 187 mM do not have such an obvious influence and furnish the expected product in 61 and 59% yields, respectively.

A time dependency study clearly showed that the best yields were achieved after 2 hours and longer reaction times did not lead to improved yields (Table 4).

**Table 4:** Time dependency of domino aldol reactions in the presence of various metal chlorides (reaction temperature: 25 °C, **5a**/**6a** in %).

Entry	Time	AlCl_3_		GaCl_3_		InCl_3_		ZrCl_4_		SnCl_4_	

		**5a**	**6a**	**5a**	**6a**	**5a**	**6a**	**5a**	**6a**	**5a**	**6a**

1	10 min	34	14	15	70	56	9	23	26	15 (20)^a^	40
2	30 min	39	11	22	61	60	10	21	20	21 (19)^a^	18
3	60 min	48	10	42	40	68	10	43	16	34 (29)^a^	17
4	120 min	66	8	70	19	87	-	68	22	36 (29)^a^	22
5	1 day	–	–	−	–	73	3	67	6	–	–
6	5 days	–	–	–	–	75	5	64	5	–	–

^a^Second diastereomer.

### Variation of enolate and aromatic aldehyde

Subsequently, various aldehydes were tested in the domino aldol reaction with propiophenone enolate in combination with different metals (Table 5). All aromatic aldehydes, even those containing strongly coordinating substituents such as the dimethylamino group, are accepted in this transformation. With anthracene-9-carbaldehyde (**3f**), however, the yields drastically decreased, most likely due to steric hindrance. Although benzaldehyde (**3a**) was not used, product **5a** appeared in the reaction of anthracene-9-carbaldehyde (**3f**) with Al and Zr metals, a finding that requires an explanation (vide infra). The NMR investigations suggest the same relative configuration of **5b**−**j** as in **5a** since coupling constants, the shift of the 2-CH_3_ group and the coupling constant *J*_(4-H, 5-H)_ agreed.

The facile formation of domino products from aromatic aldehydes proposed to use this reaction also with aromatic dialdehydes (**3j**−**l**). As anticipated the reaction proceeded smoothly with terephthalaldehyde (**3j**) giving rise to product **5j** (see Scheme 2 and Table 5), while isophthalaldehyde (**3k**) provided a mixture of isomers, which were not separable. The steric congestion in *o*-phthalaldehyde (**3l**) precluded the formation of the domino-aldol product. However, it is known that *o*-phthalaldehyde (**3l**) provides the corresponding aldol product in the presence of base [41].

**Table 5:** Reactions of various aldehydes with propiophenone metal enolate (reaction time: 2 h, reaction temperature: 25 °C).

Entry	Product	Aldehyde	Yield of **5** [%]			

			AlCl_3_	InCl_3_	SnCl_4_	ZrCl_4_

1	**5b**	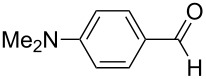 **3b**	14	47	34	40
2	**5c**	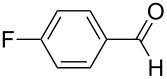 **3c**	44	94	36	60^a^
3	**5d**	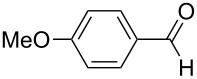 **3d**	17	72	–	45
4	**5e**	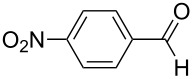 **3e**	–	50	28^a^	45
5	**5f**	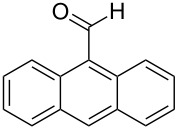 **3f**	13^b^	55	–	29^b^
6	**5g**	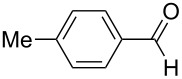 **3g**	–	62	–	66
7	**5h**	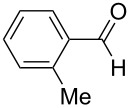 **3h**	–	50	–	51
8	**5i**	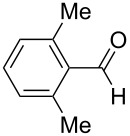 **3i**	–^c^	53	–^c^	–^c^
9	**5j**^d^	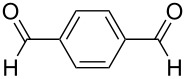 **3j**	–	35	–	30
10	**5k**	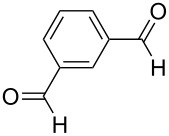 **3k**	–	–^e^	–	–^e^
11	**5l**	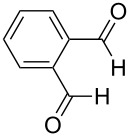 **3l**	–	–^f^	–	–^f^

^a^Ref. [37]. ^b^Additionally 10% (Al) and 20% (Zr) of **5a** are formed. ^c^The reaction was not carried out. ^d^1,4-Bis-(2,4-dimethyl-3,5-diphenyl-3,5-dihydroxytetrahydropyranyl)-benzene. ^e^Inseparable mixture. ^f^No reaction.

**Scheme 3:**
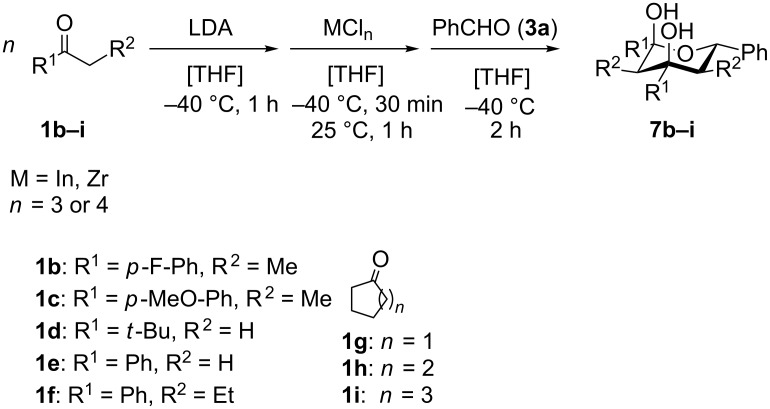
Reaction of various ketones (**1b**−**i**) with benzaldehyde (**3a**) in the presence of InCl_3_ and ZrCl_4_.

The variability in the ketone moiety proved to be rather restricted (Scheme 3). While in the case of propiophenone (**1a**) and butyrophenone (**1f**) moderate to good yields (≥50%) were obtained, the reaction with acetophenone (**1e**) did not furnish any domino aldol product at all. The only acyclic aliphatic ketone that led to the formation of the domino aldol product was pinacolone (**1d**), which gave the tetrahydro-2*H*-pyran-2,4-diol **7d** in 20% yield (Scheme 3, Table 6). The reactions of the cyclic ketones were only successful in the case of cyclohexanone (**1h**), while transformations with five- (**1g**) and seven- (**1i**) membered rings failed most likely due to strain.

**Table 6:** Variation of the ketone in the domino aldol reaction with benzaldehyde (**3a**) in the presence of InCl_3_ and ZrCl_4_ (reaction time: 2 h, reaction temperature: 25 °C).

Entry	Product	Ketone	Yield of **7** [%]		Product	Ketone	Yield of **7** [%]	
	
			InCl_3_	ZrCl_4_			InCl_3_	ZrCl_4_

1	**7b**	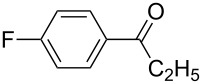 **1b**	–^a^	40	**7f**^b^	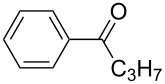 **1f**	70	50
2	**7c**	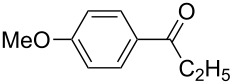 **1c**	–^a^	45	**7g**	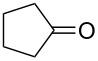 **1g**	–	–
3	**7d**	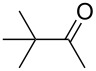 **1d**	20	–	**7h**	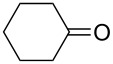 **1h**	5	5
4	**7e**	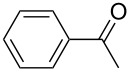 **1e**	–	–	**7i**	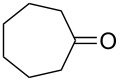 **1i**	–	–

^a^The reaction was not carried out. ^b^Ref. [42].

**Figure 2:**
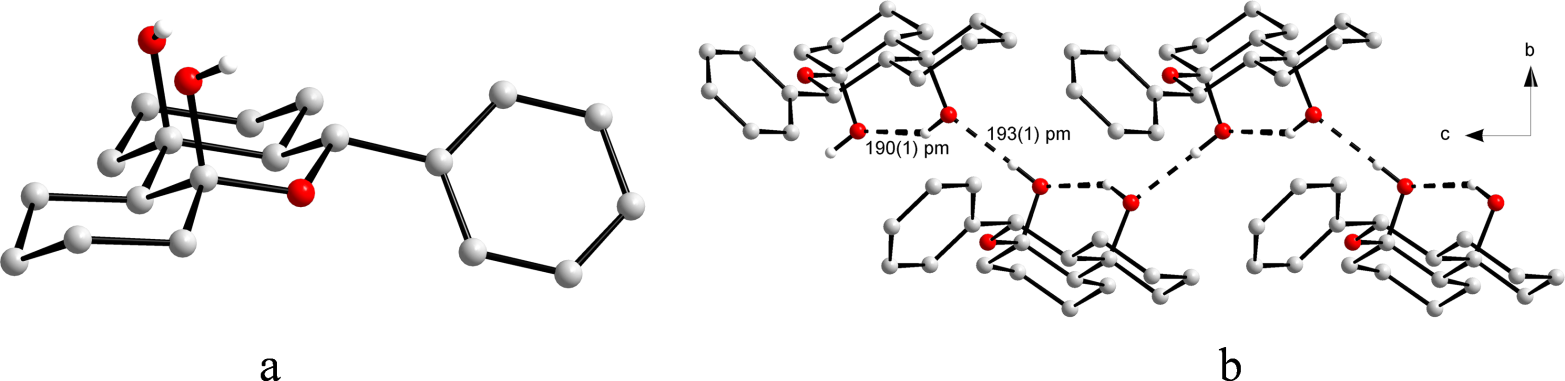
(a) Crystal structure of **7h** and (b) its arrangement in the crystal [43].

The structure of product **7h**, which precipitated from the crude mixture in crystalline form, was solved by single crystal X-ray analysis. The crystals are in the space group *P*2_1_/*c*. It forms "fibers" with alternating incorporation of the two enantiomers of **7h** and they are held together by hydrogen bonds (intramolecular: 1.90 Å, intermolecular: 1.93 Å) (Figure 2).

**Scheme 4:**
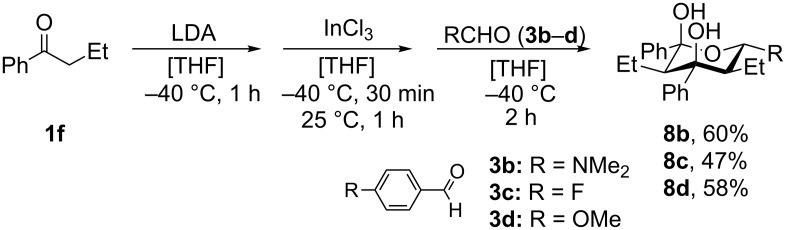
Reaction of *n*-butyrophenone (**1f**) with various aldehydes (**3b**−**d**) in presence of InCl_3_ (reaction time: 2 h, reaction temperature: 25 °C).

Various aldehydes were also probed in the domino aldol reaction with the indium enolate of butyrophenone (**1f,** Scheme 4). Reactions involving 2-furfural, cinnamaldehyde, butyraldehyde, isobutyraldehyde and 2-phenylpropanal did not provide the corresponding tetrahydro-2*H*-pyrans, while the reaction with aldehydes possessing *p*-NMe_2_ (**3b**), *p*-F (**3c**) and *p*-MeO (**3d**) substituted phenyl units worked in reasonable yields affording **8b**–**d**.

**Scheme 5:**
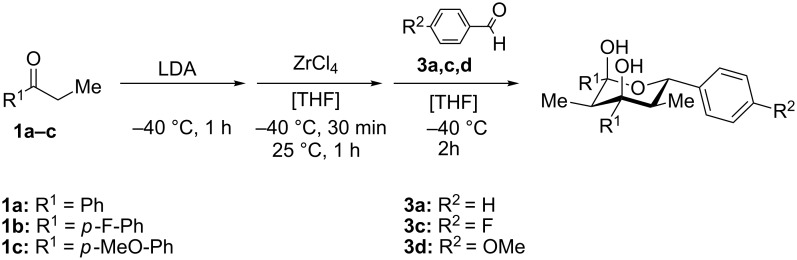
Domino aldol reactions of different aldehydes and ketones possessing *p*-H, *p*-F and *p*-MeO substituents at the phenyl units with ZrCl_4_. Products see Table 7.

The electronic influence of substituents at the aldehyde and/or ketone moiety was more systematically analyzed using series of benzaldehydes (**3a**, **3c** and **3d**) and propiophenones (**1a**–**c**) both substituted by H, OMe and F at the *para*-phenyl position. Yields increased with time as expected. The introduction of two F or MeO substituents leads to a decrease of the yield compared with **5a** (Scheme 5, Table 7). The best yields were obtained within a series in the case of ketone and aldehyde possessing a donor–acceptor situation.

**Table 7:** Time dependent domino aldol reactions of different aldehydes and ketones having H, F and MeO units at *para*-position of phenyl units using ZrCl_4_. Yields are in % and numbers in brackets are for mono aldol products (reaction time: 2 h, reaction temperature: 25 °C).

		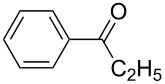			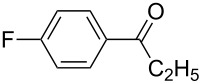			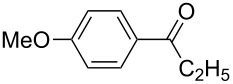		
		**1a**			**1b**			**1c**		
		
	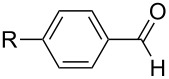 **3a–c**	H (**3a**)	F (**3c**)	MeO (**3d**)	H (**3a**)	F (**3c**)	MeO (**3d**)	H (**3a**)	F (**3c**)	MeO (**3d**)
	
Entry	Time [min]	**5a**	**5c**	**5d**	**7a**	**9a**	**9b**	**7b**	**9c**	**9d**

1	5	5 (+10)	9 (+9)	7 (+12)	5 (+15)	5 (+18)	8 (+14)	25 (+7)	26 (+11)	15 (+19)
2	10	8 (+16)	23 (+10)	10 (+15)	6 (+20)	8 (+19)	10 (+15)	28 (+8)	26 (+11)	17 (+15)
3	20	19 (+16)	27 (+8)	15 (+17)	8 (+16)	12 (+15)	14 (+17)	29 (+7)	28 (+20)	20 (+9)
4	30	27 (+18)	35 (+10)	16 (+16)	12 (+16)	15 (+19)	20 (+16)	32 (+12)	32 (+12)	25 (+11)
5	45	44 (+19)	51 (+9)	20 (+13)	19 (+19)	20 (+17)	30 (+17)	37 (+6)	36 (+17)	29 (+10)
6	60	60 (+20)	52 (+10)	35 (+12)	24 (+18)	21 (+18)	37 (+15)	40 (+6)	44 (+11)	30 (+9)
7	120	76 (+8)	62 (+7)	45 (+5)	40 (+20)	29 (+17)	54 (+14)	45 (+4)	58 (+14)	32 (+14)

### Domino aldol reaction by using a CYTOS™ microreactor

Using the CYTOS™ microreactor, a continuous reactor, the following results were obtained, which are well in agreement with the outcome of the batch experiments. Since a slow flow rate of 1 mL min^−1^ caused precipitation of the compounds and consequently blocking the reactor, flow rates from 2 mL min^−1^ onwards were used to run the reaction (Table 8). The breakdown of the yields with increasing flow rates is easily explained with the short reaction time.

**Table 8:** Reaction of benzaldehyde (**3a**) with indium propiophenone enolate in the CYTOS^TM^ Labsystem (**5a**/**6a** in %).

Entry	Flow rate [mL min^−1^]	22 °C		34 °C		47 °C		Average rxn. time [min]

		**5a**	**6a**	**5a**	**6a**	**5a**	**6a**	

1	1	−	−	−	−	−	−	60
2	2	62	12	59	10	50	14	30
3	3	63	14	58	14	48	12	20
4	5	21	12	20	9	12	6	12
5	9	20	8	22	6	25	5	6

## Discussion

The experimental results indicated that two diastereomeric tetrahydro-2*H*-pyran-2,4-diols were formed at 0 °C in the presence of gallium and at 25 °C with Sn(IV), but only one (i.e., **5a**) at elevated temperature. Such finding is indicative of thermodynamic control in the reaction. In the first step, presumably a metal di-, tri- or tetraenolate is formed based on the ratio of enolate to metal chloride. Because in principle a metal dienolate is sufficient as the nucleophilic component, additional enolate ligands may simply act as "innocent bystander ligands" in the reaction cascade. Since we have been able to obtain **5a** with GaCl_3_ in a diastereomerically pure form at 25 °C – although at low temperature a sizeable amount of 20% of a second diastereomer was formed – it is reasonable to assume a reversible formation of the metal-bound tetrahydro-2*H*-pyran-2,4-diol. Under thermodynamic control all large substituents R (methyl, phenyl) are placed in the equatorial position which leads for all metal ions excluding Ga to only 1 out of 16 possible diastereoisomers.

**Scheme 6:**
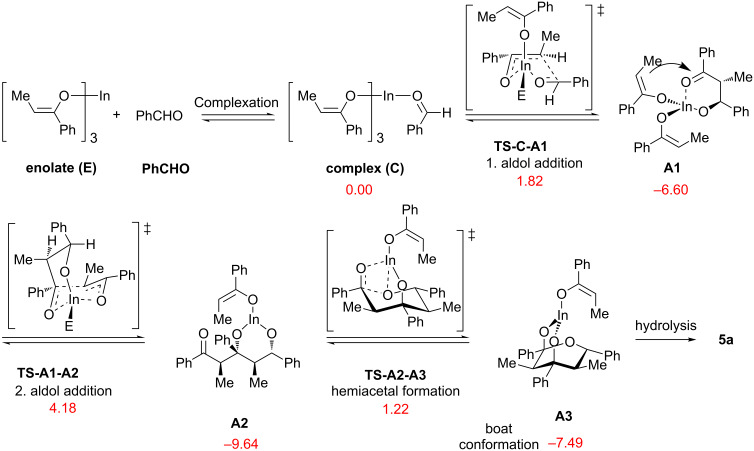
DFT calculations on the formation of **A3**, hydrolysis of which provides **5a**, at M06/6-31G(d)/LANL2DZ//B3LYP/6-31G(d)/LANL2DZ level (Δ*G*_rel_ with unscaled zpe are in kcal mol^−1^).

To shed more light on the mechanism, DFT calculations were carried out using the Gaussian 09 program [44]. Gas-phase optimization of geometries was performed by using the B3LYP [45–47] method with Pople’s split-valence 6-31G(d) basis set on C, H, O atoms and double-ζ quality basis set (LANL2DZ) [48–50] containing Hay and Wadt’s effective core potential (ECP) on hexa-coordinate indium [51] as implemented in Gaussian 09 owing to the predicted good results in our earlier work [30]. The remaining coordination sites of indium were occupied by two THF molecules. The minima and transition states of the calculated structures were verified by analyzing the harmonic vibrational frequencies, using analytical second derivatives. To predict the energies plausibly, as recommended for organometallic compounds, single point calculations with M06 functional [52] were performed using the same basis sets (Scheme 6).

Complexation of the metal enolate **E** with benzaldehyde (**PhCHO**) is followed by the exergonic first aldol addition showing a small activation barrier of 1.82 kcal mol^−1^ via a half−chair like transition state (**TS-C-A1**), which is in accord with the *anti*-selective aldol addition of titanium enolates [53–54]. **TS-C-A1** leads to the formation of *anti*-aldolate **A1**, possessing Δ*G*_rel_ of −6.60 kcal mol^−1^. In the next step, **A1** is attacked by a second enolate at higher temperature via the bicyclic transition state **TS-A1-A2** (Δ*G*_rel_ = 4.18 kcal mol^−1^) with a chair–chair conformation. In the last step, intramolecular cyclization with a relative TS energy of 1.22 kcal mol^−1^ (**TS-A2-A3**) takes place, which furnishes the metal-bound hemiacetal in a boat conformation (**A3**) with a relative free energy of −7.49 kcal mol^−1^. Hydrolysis of **A3** provides tetrahydro-2*H*-pyran-2,4-diol **5a**.

**Scheme 7:**
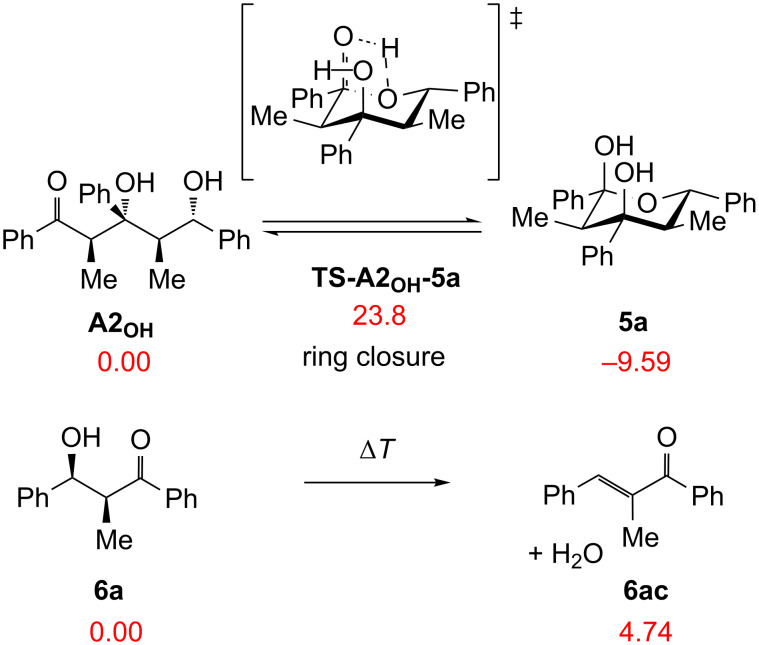
The follow-up reactions of **A2_OH_** and **6a** at M06/6-31G(d)//B3LYP/6-31G(d) level (Δ*G*_rel_ with unscaled zpe are in kcal mol^−1^).

Computationally predicted **A2** has a lower free energy than the hemiacetal **A3**, which is responsible for the formation of product **5a**. This finding suggests that the hydrolysis occurs on the stage of **A2** furnishing **A2_OH_**. Afterwards, intramolecular ring closure of **A2_OH_** with a relative activation barrier of 23.8 kcal mol^−1^ leads to **5a** in an exergonic process (Scheme 7). At higher temperature, the condensation product **6ac** emerges from the dehydration of **6a**, taking place via an irreversible reaction, which is accountable for the decrease of the yield at higher temperatures.

The mechanism depicted in Scheme 6 is in agreement with the following set of requirements with regard to the metal center: it should (a) exhibit Lewis acidity, (b) be sufficiently electropositive, and (c) display a sufficiently large ion radius so that the reaction cascade can take place in the periphery of the metal. The latter requirement is apparently prohibitive for a boron(III)-mediated reaction because the ion radius of the B^3+^ ion is very small (25 pm). In the case of tin(II), a high yield of **6a** (up to 90%) was observed but formation of **5a** was not detected which indicates a hindrance for the second aldol addition. Presumably due to the low charge density of the Sn^2+^ ions the second carbonyl function is not sufficiently activated for the last ketone–ketone–aldol step. For lanthanum and cerium and maybe even for tin(II) the size may cause problems since these ions are too big. The distance between the reactants is probably too large for a bond formation (Table 9).

**Table 9:** Effect of the properties of metals on the yields of **5a** (reaction time: 2 h).

Metal	χ^a^	Ionic radius^b^ [pm]	Charge density *Z*^2^/*r* [e^2^Å^−1^]	Yield **5a** [%]

SnCl_2_	1.72	102 (CN 2)	3.92	–
ZrCl_4_	1.22	80 (CN 5)	20.00	76
SnCl_4_	1.72	76 (CN 5)	21.05	34
TiCl_4_	1.32	56 (CN 5)	28.57	56
BCl_3_	2.01	25 (CN 4)	36.00	–
AlCl_3_	1.47	53 (CN 4)	16.98	52
GaCl_3_	1.82	61 (CN 4)	14.80	93
InCl_3_	1.49	76 (CN 4)	11.84	85
LaCl_3_	1.08	117 (CN 6)	7.69	–
CeCl_3_	1.08	115 (CN 6)	7.82	–
Sc(OTf)_3_	1.20	89 (CN 6)	10.11	–

^a^ χ: Electronegativity (According to Allred and Rochow). ^b^ CN = Coordination number [55].

**Scheme 8:**
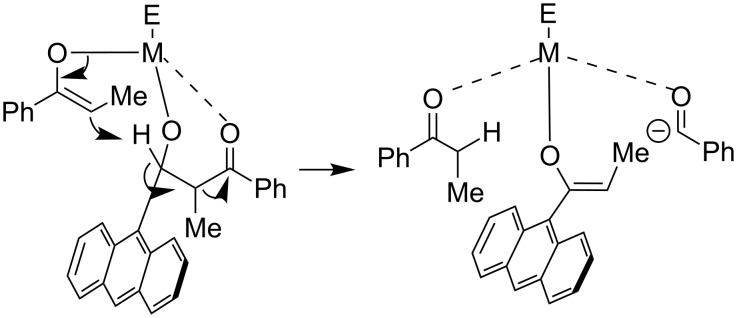
Proposed mechanism for the formation of benzaldehyde in the reaction of 9-anthracenylaldehyde (**3f**) with Zr and Al.

In the case of 9-anthracenylaldehyde (**3f**) employing Zr or Al, formation of **5a**, which was not observed in the presence of indium, is detected. Most likely the size of the anthracenyl moiety decreases the rate of the second aldol reaction or the hemiacetal formation, so that deprotonation and subsequently a retro-aldol reaction takes place (Scheme 8). The formation of a domino aldol product with two anthracenyl residues was not observed most probably due to the steric demand of the anthracenyl unit.

As illustrated in Scheme 8, the conversion of the aldehyde to the ketone moiety was also witnessed with benzaldehyde (**3a**) and 4-methoxybenzaldehyde (**3d**) in the reaction with 4'-fluoropropiophenone (**1b**) enolate and when reacting 4-methoxybenzaldehyde (**3d**) with propiophenone (**1a**) enolate. Here, there is also no interaction between the metal center and the aryl ring of the aldehyde possible. So, a competition between the second aldol or hemiacetal formation and the deprotonation should be considered.

## Conclusion

The present results demonstrate a domino aldol reaction working with several substrates and metals that is far superior to the other method with TiCp_2_ [20,22], which could only be realized with one single substrate resulting in a formal trimerization. The variations in the metal fragment are promising with regard to the development of an enantioselective version of the above reaction and further variations in the substrates. DFT calculations unveil the mechanism for the stereoselective formation of **5a**.

## Supporting Information

Experimental section, copies of ^1^H and ^13^C NMR spectra of compounds, Cartesian coordinates and CIF files of **5a** and **7h**.

File 1 Experimental section, copies of ^1^H and ^13^C NMR spectra of compounds and Cartesian coordinates.

File 2 CIF file of compound **5a**.

File 3 CIF file of compound **7h**.
