# Supplementary material for: Scope and mechanism of the highly stereoselective metal-mediated domino aldol reactions of enolates with aldehydes
Source: Beilstein J Org Chem. 2016 Apr 27;12:813–24. doi: 10.3762/bjoc.12.80 (PMC4902021; doi:10.3762/bjoc.12.80)
Supplement: File 1 — Experimental section, copies of 1H and 13C NMR spectra of compounds and Cartesian coordinates. [file Beilstein_J_Org_Chem-12-813-s001.pdf]

Supporting Information

for

Scope and mechanism of the highly stereoselective metal-mediated domino aldol reactions of enolates with aldehydes

M. Emin Cinar*, Bernward Engelen, Martin Panthöfer, Hans-Jörg Deiseroth, Jens Schlirf, and Michael Schmittel*

Address: Department Chemie-Biologie, OC1, Universität Siegen, Adolf-Reichwein-Str., D-57068 Siegen, Germany

Email: Michael Schmittel - schmittel@chemie.uni-siegen.de; M. Emin Cinar - emin.cinar@uni-siegen.de

*Corresponding author

Experimental section, copies of ^{1}H and ^{13}C NMR spectra of compounds and Cartesian coordinates

Table of contents	Page
Experimental section	s2 – s9
X-ray data of **5a** and **7h**	s10 – s11
^{1}H and ^{13}C NMR spectra	s12 – s31
Cartesian coordinates	s32 – s38

Experimental section

Starting materials were purchased from Acros Organics, Aldrich, Merck or Lancaster and used as received. Aldehydes were distilled, if necessary. Standard inert atmosphere and Schlenk techniques were employed for all reactions. THF was distilled under nitrogen directly over potassium. ^{1}H NMR and ^{13}C NMR were measured on a Bruker AC 400 (400 MHz) or Varian 600 ASC (600 MHz). All the ^{1}H NMR measurements were carried out at room temperature in $CDCl_3$ and DMSO-d_6; chemical shifts refer to tetramethylsilane. IR spectra were recorded on a Perkin-Elmer FT-IR 1605. Elemental analyses were carried out on an EA 3000 CHNS Elemental Analyzer. Uncorrected melting points were determined by using a Mettler FP5.0 melting point apparatus. The chiral column Chiralpack AD was purchased from Firma Daicel Chemical Industries. The continuous process was established using a CYTOS™ microreactor from Cellular Process Chemistry Systems Germany [1].

General procedure for the synthesis of racemic (*l,l,l,u*)-tetrahydro-2*H*-pyrans. A solution of diisopropylamine (1.26 mL, 9.00 mmol) in THF (30 mL) was treated with 3.00 mL of *n*-butyllithium (2.5 M in *n*-hexane, 7.50 mmol) at 0 °C and stirred for 15 min. After cooling down to −40 °C, propiophenone (**1a**) (1.01 mL, 7.50 mmol) was added and the mixture was stirred at −40 °C for 1 h. Then 2.5 mmol of the metal halide were added. The yellow reaction mixture was stirred for 30 min at −40 °C and for 1 h at room temperature. After that it was treated with a solution of benzaldehyde (**3a**) (250 mg, 2.50 mmol) in 30 mL of THF at different reaction temperatures and the reaction mixture was stirred for 2 h at the corresponding reaction temperatures (see Tables in manuscript). It was quenched with saturated aqueous ammonium chloride solution (50 mL) and the aqueous layer was extracted three times with diethyl ether (30 mL). The combined organic layers were washed with brine and dried over Na_2SO_4.

In case of the following metals the general procedure was modified: a) $Ti(OiPr)_2Cl_2$: 700 μL of diisopropylamine (5.50 mmol), 2.00 mL of *n*-BuLi (2.5 M in *n*-hexane, 5.50 mmol) and 660 μL of propiophenone (**1a**) (5.50 mmol) in THF (30 mL) were used. The addition of the aldehyde solution was done at reflux temperature. b) $ZrCl_4$: The neat aldehyde was added. The overall amount of THF was 30 mL. c) $SnCl_4$: 720 μL of diisopropylamine (5.10 mmol), 2.00 mL of *n*-BuLi (2.5 M in *n*-hexane, 5.00 mmol) and 660 μL of propiophenone (5.00 mmol) were reacted.

---

1. Taghavi-Moghadam, S.; Kleemann, A.; Golbig, K. G. *Org. Proc. Res. Dev.* **2001**, *5*, 652−658; for additional information: www.cpc-net.com.

3,5-Dimethyl-2,4,6-triphenyltetrahydro-2*H*-pyran-2,4-diol (5a). $AlCl_3$ (333 mg, 2.50 mmol), $InCl_3$ (555 mg, 2.50 mmol), $Ti(OiPr)_2Cl_2$ (593 mg, 2.50 mmol), $ZrCl_4$ (583 mg, 2.50 mmol) and $SnCl_4$ (326 mg, 1.25 mmol) were added in the different experiments. After removal of the solvent the crude product was washed with *n*-hexane. Yields: with $AlCl_3$ 618 mg (1.65 mmol, 64%), with $InCl_3$ 814 mg (2.18 mmol, 85%), with $Ti(OiPr)_2Cl_2$ 843 mg (2.25 mmol, 50%), with $ZrCl_4$ 711 mg (1.90 mmol, 76%) and with $SnCl_4$ 318 mg (900 μmol, 36%) of **5a** obtained as a colorless solid. The results of the spectroscopic measurements are well in agreement with the reported ones [2].

3,5-Dimethyl-6-(4-(*N*,*N*-dimethylamino)phenyl)-2,4-diphenyltetrahydro-2*H*-pyran-2,4-diol (5b). $AlCl_3$ (333 mg, 2.50 mmol), $InCl_3$ (555 mg, 2.50 mmol), $Ti(OiPr)_2Cl_2$ (593 mg, 2.50 mmol), $ZrCl_4$ (583 mg, 2.50 mmol) and $SnCl_4$ (326 mg, 1.25 mmol) were added in the different experiments. 374 mg of 4-(*N*,*N*-dimethylamino)benzaldehyde (**3b**) (2.50 mmol) was added. The crude product obtained after work up was recrystallized from ethanol furnishing with $AlCl_3$ 146 mg (350 μmol, 14%) with $InCl_3$ 491 mg (1.18 mmol, 47%) with $ZrCl_4$ 418 mg (1.00 mmol, 40%) and with $SnCl_4$ 355 mg (854 μmol, 34%) of **5b** as a colorless solid. Spectroscopic measurements are well in alignment with the reported literature values [2].

6-(4-Fluorophenyl)-3,5-dimethyl-2,4-diphenyltetrahydro-2*H*-pyran-2,4-diol (5c). $AlCl_3$ (333 mg, 2.50 mmol), $InCl_3$ (555 mg, 2.50 mmol), $Ti(OiPr)_2Cl_2$ (593 mg, 2.50 mmol), $ZrCl_4$ (583 mg, 2.50 mmol) and $SnCl_4$ (326 mg, 1.25 mmol) were added in the different experiments. 260 μL of 4-fluorobenzaldehyde (**3c**) (2.50 mmol) was introduced. The crude product was recrystallized from ethanol furnishing with $AlCl_3$ 432 mg (1.10 mmol, 44%) with $InCl_3$ 922 mg (2.35 mmol, 94%) with $ZrCl_4$ 589 mg (1.50 mmol, 60%) and with $SnCl_4$ 177 mg (450 μmol, 36%) of **5c** as a colorless solid. Spectroscopic measurements are well in alignment with the reported literature values [2].

6-(4-Methoxyphenyl)-3,5-dimethyl-2,4-diphenyltetrahydro-2*H*-pyran-2,4-diol (5d). $AlCl_3$ (333 mg, 2.50 mmol), $InCl_3$ (555 mg, 2.50 mmol), $Ti(OiPr)_2Cl_2$ (593 mg, 2.50 mmol) or $ZrCl_4$ (583 mg, 2.50 mmol) were used in the different experiments. Then, 300 μL of 4-methoxybenzaldehyde (**3d**) (2.5 mmol) was introduced. Recrystallization of the crude product from ethanol furnished with $AlCl_3$ 172 mg (430 μmol, 17%), with $InCl_3$ 728 mg (1.80 mmol, 72%) and with $ZrCl_4$ 455 mg (1.13 mmol, 45%) of **5d** as a colorless solid. Spectroscopic measurements are well in alignment with the reported literature values [3].

2. Haeuseler, A.; Henn, W.; Schmittel, M. *Synthesis*, **2003**, *16*, 2576−2589.
3. Schmittel, M.; Ghorai, M. K. *Synlett*, **2001**, *12*, 1992−1994.

3,5-Dimethyl-6-(4-nitrophenyl)-2,4-diphenyltetrahydro-2*H*-pyran-2,4-diol (5e). $ZrCl_4$ (583 mg, 2.50 mmol) as a coordination metal, and 4-nitrobenzaldehyde (**3e**) (378 mg, 2.50 mmol) were used. The crude product was purified by crystallization from *n*-hexane furnishing 472 mg (1.13 mmol, 45%) of **5e**. Spectroscopic measurements are well in alignment with the reported literature values [2].

6-Anthracen-9-yl-3,5-dimethyl-2,4-diphenyltetrahydro-2*H*-pyran-2,4-diol (5f). $AlCl_3$ (333 mg, 2.50 mmol), $InCl_3$ (555 mg, 2.50 mmol) and $ZrCl_4$ (583 mg, 2.50 mmol) were added in the different experiments. 524 mg of 9-anthracenecarbaldehyde (**3f**) (2.50 mmol) was introduced. The crude product was recrystallized from *n*-pentane affording with $AlCl_3$ 154 mg (320 μmol, 13%), with $InCl_3$ 652 mg (1.38 mmol, 55%) and with $ZrCl_4$ 344 mg (725 μmol, 29%) of **5f** as a yellow solid. Spectroscopic measurements match well with the reported literature values [3].

3,5-Dimethyl-2,4-diphenyl-6-(*p*-tolyl)tetrahydro-2*H*-pyran-2,4-diol (5g). $InCl_3$ (555 mg, 2.50 mmol) and $ZrCl_4$ (583 mg, 2.50 mmol) were added in the different experiments. 295 μL of *p*-tolylaldehyde (**3g**) (2.50 mmol) was added. The crude product was purified by crystallization from *n*-hexane resulting in with $InCl_3$ 602 mg (1.55 mmol, 62%) and with $ZrCl_4$ 641 mg (1.65 mmol, 66%) of **5g** as a colorless solid. Mp. 174 °C. ^{1}H NMR (400 MHz, $CDCl_3$): δ 0.44 (d, J = 6.8 Hz, 3H), 0.59 (d, J = 7.2 Hz, 3H), 2.31−2.39 (m, 5H), 3.91 (s, 1H), 3.99 (d, J = 1.6 Hz, 1H), 5.01 (d, J = 10.4 Hz, 1H), 7.17−7.41 (m, 11H), 7.66−7.73 ppm (m, 3H). ^{13}C NMR (100 MHz, $CDCl_3$): δ 9.5, 10.8, 21.2, 46.7, 47.9, 75.4, 78.1, 101.5, 123.7, 125.9, 126.3, 126.4, 127.5, 127.7, 127.9, 128.1, 128.2, 129.0, 137.6, 137.7, 143.4, 144.3 ppm. IR (KBr): 3431, 3045, 2973, 2919, 1516, 1447, 1384, 1226, 1061, 1037, 1017, 968, 925, 820, 772, 750, 699, 550 cm^{-1}. Anal. Calcd for $C_{26}H_{28}O_3$ (388.20 g mol^{-1}): C 80.38, H 7.26; Found: C 80.45, H 7.11.

3,5-Dimethyl-2,4-diphenyl-6-(*o*-tolyl)tetrahydro-2*H*-pyran-2,4-diol (5h). $InCl_3$ (555 mg, 2.50 mmol) and $ZrCl_4$ (583 mg, 2.50 mmol) were added in the different experiments. 287 μL of *o*-tolylaldehyde (**3h**) (2.50 mmol) was introduced. The crude product was recrystallized from *n*-hexane providing with $InCl_3$ 486 mg (1.25 mmol, 50%) and with $ZrCl_4$ 497 mg (1.28 mmol, 51%) of **5h** as a colorless solid. Mp. 172 °C. ^{1}H NMR (400 MHz, $CDCl_3$): δ 0.44 (d, J = 6.8 Hz, 3H), 0.59 (d, J = 6.8 Hz, 3H), 2.31−2.39 (m, 5H), 3.93 (d, J = 3.2 Hz, 1H), 4.01 (s, 1H), 5.02 (d, J = 10.4 Hz, 1H), 7.17−7.24 (m, 4H), 7.29−7.41 (m, 7H), 7.66−7.73 ppm (m, 3H). ^{13}C NMR (100 MHz, $CDCl_3$): δ 9.5, 10.8, 21.2, 46.7, 47.9, 75.4, 78.1, 101.5, 123.7, 123.8, 126.0, 126.3, 126.4, 127.6, 127.9, 128.0, 128.2, 129.0, 137.6, 137.8, 143.5, 144.3 ppm. IR (KBr): 3427, 3055, 3027, 2973, 2938, 2918, 1516, 1497, 1447, 1384, 1255, 1226, 1157, 1061, 1037, 1017, 968, 820, 772, 750, 699 cm^{-1}. Anal. Calcd for $C_{26}H_{28}O_3$ (388.20 g mol^{-1}): C 80.38, H 7.26; Found: C 80.53, H 6.79.

6-(2,6-Dimethylphenyl)-3,5-dimethyl-2,4-diphenyltetrahydro-2*H*-pyran-2,4-diol (5i). $InCl_3$ (555 mg, 2.50 mmol) and 333 mg of 2,6-dimethylbenzaldehyde (**3i**) (2.50 mmol) were reacted. The crude product

was purified by crystallization from *n*-hexane giving 533 mg (1.33 mmol, 53%) of **5i** as a colorless solid. Mp. 108 °C. ^{1}H NMR (400 MHz, $CDCl_3$): δ 0.46 (d, J = 6.8 Hz, 3H), 0.58 (d, J = 6.8 Hz, 3H), 2.38−2.46 (m, 4H), 2.88 (s, 3H), 2.90−2.98 (m, 1H), 4.00 (s, 1H), 4.04 (s, 1H), 5.78 (d, J = 11.2 Hz, 1H), 6.95−7.06 (m, 3H), 7.21−7.43 (m, 7H), 7.66−7.72 ppm (m, 3H). ^{13}C NMR (100 MHz, $CDCl_3$): δ 9.6, 10.1, 21.6, 21.7, 42.9, 47.9, 70.6, 78.5, 101.8, 123.6, 125.8, 126.5, 127.5, 128.0, 128.2, 128.3, 130.5, 135.3, 137.2, 137.3, 143.3, 144.4 ppm. IR (KBr): 3420, 3048, 2977, 2942, 1447, 1381, 1228, 1115, 1056, 1030, 993, 964, 923, 771, 747, 700, 615 cm^{-1}. Anal. Calcd for $C_{27}H_{30}O_3$ (402.53 g mol^{-1}) C 80.56, H 7.51; Found: C 80.62, H 7.68.

1,4-Bis-(2,4-dimethyl-3,5-diphenyl-3,5-dihydroxytetrahydropyranyl)-benzene (5j). The amounts of the ketone and the coordination metals were doubled. Terephthalic aldehyde (**3j**) (335 mg, 2.50 mmol) was added. The crude product was purified by recrystallization from ethanol furnishing with $InCl_3$ 342 mg (850 μmol, 35%) and with $ZrCl_4$ 305 mg (304 μmol, 30%) of **5j** as a colorless solid. Mp. 180 °C. ^{1}H NMR (400 MHz, DMSO-d$_6$): δ 0.28 (d, J = 6.4 Hz, 3H), 0.29 (d, J = 6.8 Hz, 3H), 0.36 (d, J = 7.2 Hz, 3H), 0.37 (d, J = 6.8 Hz, 3H), 2.36−2.41 (m, 4H), 4.92 (d, J = 10.4 Hz, 1H), 5.01 (d, J = 10.4 Hz, 1H), 5.41 (s, 1H), 5.42 (s, 1H), 7.16−7.20 (m, 2H), 7.29−7.40 (m, 13H), 7.55−7.74 (m, 9H), 7.97 ppm (d, J = 8.4 Hz, 2H). ^{13}C NMR (100 MHz, DMSO-d$_6$): δ 9.7, 10.8, 45.9, 46.8, 74.8, 77.5, 100.7, 124.2, 126.1, 126.3, 127.4, 127.5, 127.6, 127.9, 128.0, 128.7, 132.9, 141.4, 143.9, 144.9 ppm. IR (KBr): 3399, 3058, 3028, 2976, 2939, 1680, 1600, 1447, 1384, 1225, 1061, 1033, 1019, 996, 967, 833, 772, 745, 700, 616 cm^{-1}. Anal. Calcd for $C_{44}H_{46}O_6$ (670.83 g mol^{-1}): C 78.78, H 6.91; Found: C 78.45, H 7.25.

2,4-Bis-(4-fluorophenyl)-3,5-dimethyl-6-phenyltetrahydro-2*H*-pyran-2,4-diol (7b). 4-Fluoropropiophenone (**1b**) (1.41 g, 7.5 mmol) was used as a ketone and $ZrCl_4$ (583 mg, 2.50 mmol) as a coordination metal. Benzaldehyde (**3a**) (250 mg, 2.50 mmol) was introduced. The crude product was recrystallized from ethanol affording 164 mg (1.00 mmol, 40%) of **7b** as a colorless solid. Mp. 187 °C. ^{1}H NMR (400 MHz, $CDCl_3$): δ 0.43 (d, J = 6.8 Hz, 3H), 0.58 (d, J = 7.1 Hz, 3H), 2.24−2.34 (m, 2H), 3.78 (d, J = 1.2 Hz, 1H), 3.93 (s, 1H), 5.02 (d, J = 10.4 Hz, 1H), 7.02−7.16 (m, 5H), 7.30−7.49 (m, 5H), 7.62−7.69 ppm (m, 3H). ^{13}C NMR (100 MHz, DMSO-d$_6$): δ 9.6, 10.7, 45.7, 46.7, 74.9, 77.2, 100.5, 113.8 (d, J = 20 Hz), 114.1 (d, J = 21 Hz), 114. 7 (d, J = 17 Hz), 115.0 (d, J = 17 Hz), 126.3, 127.4, 127.9, 128.0, 128.4, 128.5, 129.8 (d, J = 8.1 Hz), 140.3 (d, J = 2.8 Hz), 141.1 (d, J = 3.0 Hz), 141.2, 159.9 (d, J = 94 Hz), 162.3 ppm (d, J = 95 Hz). IR (KBr): 3428, 2975, 2635, 1615, 1509, 1385, 1292, 1225, 1146, 1072, 1021, 1005, 977, 821, 563 cm^{-1}. Anal. Calcd for $C_{25}H_{24}F_2O_3$ (410.45 g mol^{-1}): C 73.16, H 5.89; Found: C 73.41, H 5.72.

2,4-Bis-(4-methoxyphenyl)-3,5-dimethyl-6-phenyltetrahydro-2*H*-pyran-2,4-diol (7c). 4-Methoxypropiophenone (**1c**) (1.23 g, 7.5 mmol) was used as a ketone and $ZrCl_4$ (583 mg, 2.50 mmol) as a

coordination metal. Benzaldehyde (**3a**) (250 mg, 2.50 mmol) was added. The crude product was purified by recrystallization from ethanol providing 491 mg (1.13 mmol, 45%) of **7c** as a colorless solid. Mp. 142 °C. ^{1}H NMR (400 MHz, DMSO-d$_6$): δ 0.28 (d, J = 6.8 Hz, 3H), 0.37 (d, J = 7.2 Hz, 3H), 2.25−2.35 (m, 2H), 3.72 (s, 3H), 3.73 (s, 3H), 4.88 (d, J = 10.4 Hz, 1H), 5.34 (s, 1H), 6.85−6.92 (m, 4H), 7.22−7.38 (m, 5H), 7.44−7.56 ppm (m, 5H). ^{13}C NMR (100 MHz, DMSO-d$_6$): δ 9.7, 10.8, 45.9, 47.1, 54.9, 55.0, 74.9, 77.3, 100.7, 112.2, 112.7, 113.4, 113.9, 125.2, 127.4, 127.5, 127.6, 127.9, 128.0, 128.9, 136.4, 136.9, 141.5, 157.4, 158.6 ppm. IR (KBr): 3424, 2975, 2931, 2846, 1612, 1505, 1375, 1314, 1238, 1051, 1029, 1010, 932, 829, 535 cm^{-1}. Anal. Calcd for $C_{27}H_{30}O_5$ (434.52 g mol^{-1}): C 74.63, H 6.96; Found: C 74.75, H 6.80.

2,4-Di-*tert*-butyl-6-phenyltetrahydro-2*H*-pyran-2,4-diol (7d). The mixture possessing $InCl_3$ (555 mg, 2.50 mmol) and 936 µL (7.50 mmol) of pinacolone (**1d**) was reacted with a solution of benzaldehyde (**3a**) (250 mg, 2.50 mmol) in THF (30 mL). The crude product was purified by recrystallization from ethanol leading to 152 mg (499 µmol, 20%) of **7d** as a colorless solid. Mp. 133 °C. ^{1}H-NMR (DMSO-d$_6$, 600 MHz): δ = 0.86 (s, 9H), 0.98 (s, 9H), 1.36 (dd, J = 13.2 and 13.2 Hz, 1H), 1.59 (dd, J = 13.2 and 1.2 Hz, 1H), 1.65 (d, J = 13.2 Hz, 1H), 1.82 (d, J = 13.2 Hz, 1H), 5.02 (dd, J = 11.4 and 1.8 Hz, 1H), 5.38 (s, 1H), 6.40 (s, 1H), 7.22−7.25 (m, 1H), 7.32−7.36 ppm (m, 4H). ^{13}C-NMR (DMSO-d$_6$, 150 MHz): δ = 24.5, 24.7, 30.4, 37.5, 38.7, 38.9 67.0, 75.0, 100.4, 125.4, 126.6, 128.0, 143.9 ppm. IR [KBr]: 3348, 2967, 2721, 2488, 1602, 1467, 1370, 1252, 1218, 1143, 1086, 1018, 974, 887, 698, 551 cm^{-1}. Anal. Calcd for $C_{19}H_{30}O_3$ (306.44 g mol^{-1}): C 74.47, H 9.87; Found: C 74.21, H 9.87.

3,5-Diethyl-2,4,6-triphenyltetrahydro-2*H*-pyran-2,4-diol (7f). $InCl_3$ (555 mg, 2.50 mmol) and 1.10 mL (7.50 mmol) of *n*-butyrophenone (**1f**) were used as a coordination metal and a ketone, respectively. A solution of benzaldehyde (**3a**) (250 mg, 2.50 mmol) in THF (30 mL) was introduced. The crude product was recrystallized from ethanol resulting in 704 mg (1.75 mmol, 70%) of **7f**. Spectroscopic measurements are well in alignment with the reported literature values [2−8].

6-Phenyldodecahydro-1*H*-dibenzo[*b*,*d*]pyran-4a,10a-diol (7h). $InCl_3$ (555 mg, 2.50 mmol) and $ZrCl_4$ (583 mg, 2.50 mmol) were added in the different experiments 775 µL (7.50 mmol) of cyclohexanone was introduced. A solution of benzaldehyde (**3a**) (250 mg, 2.50 mmol) in THF (30 mL) was added. The crude product was recrystallized from ethanol resulting in with both coordination metals 37.8 mg (125 µmol, 5%) of **7h**.

---

4. Schmittel, M.; Burghart, A.; Malisch, W.; Reising, J.; Söllner, R. *J. Org. Chem.* **1998**, *63*, 396−400.
5. Schmittel, M.; Burghart, A.; Werner, H.; Laubender, M.; Söllner, R. *J. Org. Chem.* **1999**, *64*, 3077−3085.
6. Schmittel, M.; Ghorai, M. K.; Haeuseler, A.; Henn, W.; Koy, T.; Söllner, R. *Eur. J. Org. Chem.* **1999**, 2007−2010.
7. Schmittel, M.; Haeuseler, A.; Nilges, T.; Pfitzner, A. *Chem. Commun.* **2003**, 34−35.
8. Drauz, K.; Kurt, G.; Schmittel, M.; Söllner, R. Deutsches Patent- und Markenamt, 11 May **2000**, DE 19911 198 A1.

6-(4-(*N*,*N*-Dimethylamino)phenyl)-3,5-diethyl-2,4-diphenyltetrahydro-2*H*-pyran-2,4-diol (8b). The mixture containing $InCl_3$ (555 mg, 2.50 mmol) and 1.10 mL (7.50 mmol) of *n*-butyrophenone (**1f**) was treated with a solution of 4-(*N*,*N*-dimethylamino)benzaldehyde (**3b**) (375 mg, 2.50 mmol) in THF (30 mL). The crude product was recrystallized from ethanol furnishing 670 mg (1.50 mmol, 60%) of **8b**. Mp. 151 °C. ^{1}H NMR (400 MHz, DMSO-d$_6$): δ –0.27 (t, J = 7.6 Hz, 3H), –0.07 (t, J = 7.6 Hz, 3H), 0.78−0.88 (m, 1H), 1.03−1.14 (m, 3H), 2.09−2.19 (m, 2H), 2.87 (s, 6H), 4.83 (d, J = 10.4 Hz, 1H), 5.49 (s, 1H), 6.70 (d, J = 8.4 Hz, 2H), 7.18 (t, J = 7.6 Hz, 1H), 7.27−7.40 (m, 8H), 7.52 (t, J = 8.0 Hz, 1H), 7.64−7.69 ppm (m, 3H). ^{13}C NMR (100 MHz, DMSO-d$_6$): δ 13.7, 14.5, 17.8, 19.3, 40.1, 52.4, 54.0, 74.1, 78.7, 100.6, 111.7, 125.4, 126.0, 126.4, 127.4, 127.5 (2C), 128.8, 128.9, 144.1, 144.9, 149.9 ppm. IR (KBr): 3412, 3029, 2958, 2869, 1614, 1589, 1448, 1376, 1348, 1226, 1177, 1105, 1077, 1030, 944, 833, 791, 758, 702, 554 cm^{-1}. Anal. Calcd for $C_{29}H_{35}NO_3$ (445.59 g mol^{-1}) C 78.17, H 7.92; Found: C 78.45, H 7.65.

3,5-Diethyl-6-(4-fluorophenyl)-2,4-diphenyltetrahydro-2*H*-pyran-2,4-diol (8c). The mixture possessing $InCl_3$ (555 mg, 2.50 mmol) and 1.10 mL (7.50 mmol) of *n*-butyrophenone (**1f**) was reacted with a solution of 4-fluorobenzaldehyde (**3c**) (260 µL, 2.50 mmol) in THF (30 mL). The crude product was purified by recrystallization from ethanol leading to 498 mg (1.18 mmol, 47%) of **8c**. Mp. 154 °C. ^{1}H NMR (400 MHz, $CDCl_3$): δ –0.15 (t, J = 7.6 Hz, 3H), 0.00 (t, J = 7.6 Hz, 3H), 0.89−1.00 (m, 1H), 1.11−1.19 (m, 1H), 1.27−1.34 (m, 2H), 2.08−2.13 (m, 2H), 3.96 (s, 1H), 4.02 (s, 1H), 5.07 (d, J = 10.8 Hz, 1H), 7.04−7.08 (m, 1H), 7.23−7.39 (m, 7H), 7.48−7.52 (m, 2H), 7.63−7.69 (m, 2H), 7.74−7.77 ppm (m, 1H). ^{13}C NMR (100 MHz, $CDCl_3$): δ 13.7, 14.5, 17.9, 19.3, 53.6, 55.1, 74.6, 79.4, 101.5, 115.2 (d, J = 21 Hz), 124.8, 125.8, 126.6, 127.8, 128.1, 128.2, 129.5 (d, J = 8.2 Hz), 136.6 (d, J = 12 Hz), 143.1, 143.8, 163.7 ppm. IR (KBr): 3690, 3414, 3032, 2967, 2872, 1605, 1511, 1448, 1383, 1312, 1222, 1192, 1132, 1092, 1076, 1045, 1013, 837, 782, 756, 701, 619, 545, 529 cm^{-1}. Anal. Calcd for $C_{27}H_{29}FO_3$ (420.52 g mol^{-1}): C 77.12, H 6.95; Found: C 77.45, H 6.65.

3,5-Diethyl-6-(4-methoxyphenyl)-2,4-diphenyltetrahydro-2*H*-pyran-2,4-diol (8d). $InCl_3$ (555 mg, 2.50 mmol) and 1.10 mL (7.50 mmol) of *n*-butyrophenone (**1f**) were chosen as a coordination metal and a ketone, respectively. A solution of 4-methoxybenzaldehyde (**3d**) (300 µL, 2.50 mmol) in THF (30 mL) was introduced. The crude product was recrystallized from ethanol furnishing 631 mg (1.46 mmol, 58%) of **8d**. Mp. 134 °C. ^{1}H NMR (400 MHz, $CDCl_3$): δ –0.15 (t, J = 7.6 Hz, 3H), 0.02 (t, J = 7.6 Hz, 3H), 0.90−1.01 (m, 1H), 1.11−1.21 (m, 1H), 1.26−1.36 (m, 1H), 2.09−2.16 (m, 2H), 3.79 (s, 3H), 3.97 (s, 1H), 4.06 (s, 1H), 5.03 (d, J = 10.8 Hz, 1H), 6.89 (d, J = 8.6 Hz, 2H), 7.22−7.46 (m, 7H), 7.44 (d, J = 8.6 Hz, 2H), 7.66 (d, J = 7.2 Hz, 2H), 7.76−7.78 (m, 1H). ^{13}C NMR (100 MHz, $CDCl_3$): δ 13.7, 14.6, 17.9, 19.5, 53.5, 55.1, 55.2, 74.8, 79.4, 101.5, 113.7, 124.9, 125.9, 126.6, 127.8, 128.0, 129.1, 132.9, 140.6, 143.4,

144.0, 159.3 ppm. IR (KBr): 3412, 3089, 3062, 3031, 2963, 2930, 2906, 2872, 1654, 1602, 1497, 1448, 1388, 1312, 1273, 1238, 1225, 1190, 1146, 1130, 1077, 1046, 1029, 755, 738, 700, 553 cm^{-1}. Anal. Calcd for $C_{28}H_{32}O_4$ (432.55 g mol^{-1}): C 77.75, H 7.46; Found: C 78.05, H 7.65.

2,4,6-Tris-(4-fluorophenyl)-3,5-dimethyltetrahydro-2*H*-pyran-2,4-diol (9a). 4-Fluoropropiophenone (**1b**) (1.41 g, 7.5 mmol) was used as a ketone and $ZrCl_4$ (583 mg, 2.50 mmol) was chosen as a coordination metal. 4-Fluorobenzaldehyde (**3c**) (310 mg, 2.50 mmol) was added. The crude product was recrystallized from ethanol leading to formation of 311 mg (725 µmol, 29%) of **9a** as a colorless solid. Mp. 200 °C. ^1H NMR (400 MHz, DMSO-d_6): δ 0.27 (d, J = 6.8 Hz, 3H), 0.35 (d, J = 6.8 Hz, 3H), 2.34−2.42 (m, 2H), 4.91 (d, J = 10.4 Hz, 1H), 5.40 (s, 1H), 7.11−7.21 (m, 6H), 7.43 (s, 1H), 7.60−7.70 ppm (m, 6H). ^{13}C NMR (100 MHz, DMSO-d_6): δ 9.6, 10.7, 45.7, 46.7, 74.2, 77.3, 100.5, 113.8 (d, J = 21 Hz), 114.1 (d, J = 21 Hz), 114.8 (d, J = 21 Hz), 126.3 (d, J = 4.8 Hz), 128.2 (d, J = 8.2 Hz), 128.4 (d, J = 8.1 Hz), 129.8 (d, J = 7.9 Hz), 137.5 (d, J = 2.7 Hz), 140.2 (d, J = 2.6 Hz), 141.0 (d, J = 2.8 Hz), 159.5, 160.4 (d, J = 7.9 Hz), 161.9, 162.8 ppm (d, J = 8.1 Hz). IR (KBr): 3426, 2977, 2640, 1604, 1511, 1385, 1295, 1219, 1156, 1093, 1060, 1036, 1016, 966, 838, 815, 801, 557, 532 cm^{-1}. Anal. Calcd for $C_{25}H_{23}F_3O_3$ (428.44 g mol^{-1}): C 70.08, H 5.41; Found: C 70.18, H 5.45.

2,4-Bis-(4-fluorophenyl)-6-(4-methoxyphenyl)-3,5-dimethyltetrahydro-2*H*-pyran-2,4-diol (9b). The reaction mixture containing 4-fluoropropiophenone (**1b**) (1.41 g, 7.5 mmol) and $ZrCl_4$ (583 mg, 2.50 mmol) was reacted with 4-methoxybenzaldehyde (**3d**) (411 mg, 2.50 mmol). The crude product was purified by crystallization from ethanol furnishing 595 mg (1.35 mmol, 54%) of **9b** as a colorless solid. Mp. 144 °C. ^1H NMR (400 MHz, DMSO-d_6): δ 0.26 (d, J = 6.8 Hz, 3H), 0.36 (d, J = 7.2 Hz, 3H), 2.23−2.40 (m, 2H), 3.74 (s, 3H), 4.84 (d, J = 10.4 Hz, 1H), 5.39 (s, 1H), 6.91 (d, J = 8.8 Hz, 2H), 7.12−7.19 (m, 4H), 7.39 (s, 1H), 7.47 (d, J = 8.8 Hz, 2H), 7.54−7.69 ppm (m, 4H). ^{13}C NMR (100 MHz, DMSO-d_6): δ 9.6, 10.7, 45.8, 46.7, 55.0, 74.4, 77.3, 100.4, 109.2, 113.4, 113.8 (d, J = 20 Hz), 114.1 (d, J = 21 Hz), 114.7 (d, J = 15 Hz), 114.9 (d, J = 15 Hz), 126.3 (d, J = 6.5 Hz), 128.2 (d, J = 7.5 Hz), 128.4 (d, J = 8.2 Hz), 129.0, 129.8 (d, J = 8.2 Hz), 133.3, 140.3 (d, J = 2.3 Hz), 141.6 (d, J = 2.6 Hz), 158.7, 160.0 (d, J = 94 Hz), 162.8 ppm (d, J = 95 Hz). IR (KBr): 3400, 3064, 2972, 2937, 2901, 1603, 1511, 1458, 1385, 1302, 1248, 1220, 1156, 1038, 965, 835, 814, 670, 640, 558, 526 cm^{-1}. Anal. Calcd for $C_{26}H_{26}F_2O_4$ (440.48 g mol^{-1}): C 70.90, H 5.95; Found: C 70.68, H 5.87.

6-(4-Fluorophenyl)-2,4-bis-(4-methoxyphenyl)-3,5-dimethyltetrahydro-2*H*-pyran-2,4-diol (9c). The reaction mixture with *p*-methoxypropiophenone (**1c**) (1.23 g, 7.5 mmol) and $ZrCl_4$ (583 mg, 2.50 mmol) was treated with 4-fluorobenzaldehyde (**3c**) (310 mg, 2.50 mmol). The crude product was recrystallized from ethanol giving 656 mg (1.45 mmol, 58%) of **9c** as a colorless solid. Mp. 148 °C. ^1H NMR (400 MHz, DMSO-d_6): δ 0.28 (d, J = 6.8 Hz, 3H), 0.37 (d, J = 7.2 Hz, 3H), 2.26−2.34 (m, 2H), 3.72 (s, 3H),

3.74 (s, 3H), 4.90 (d, $J = 10.4$ Hz, 1H), 5.33 (s, 1H), 6.86 (d, $J = 8.8$ Hz, 2H), 6.90 (d, $J = 8.8$ Hz, 2H), 7.16−7.20 (m, 2H), 7.26 (s, 1H), 7.33−7.62 ppm (m, 6H). ^{13}C NMR (100 MHz, DMSO-d$_6$): δ 9.7, 10.7, 46.0, 47.1, 54.9, 55.0, 74.2, 77.3, 100.7, 112.1, 112.7, 113.3, 113.9, 114.7 (d, $J = 21$ Hz), 125.2, 127.5, 128.9, 129.8 (d, $J = 7.9$ Hz), 136.3, 136.9, 137.8 (d, $J = 2.9$ Hz), 157.4, 158.6, 160.3, 162.7 ppm. IR (KBr): 3417, 2982, 2941, 2836, 1611, 1511, 1385, 1294, 1248, 1175, 1061, 1037, 1015, 931, 825, 800, 535 cm^{-1}. Anal. Calcd for $C_{27}H_{29}FO_5$ (452.51 g mol^{-1}): C 71.66, H 6.46; Found: C 71.47, H 6.37.

2,4,6-Tris-(4-methoxyphenyl)-3,5-dimethyltetrahydro-2*H*-pyran-2,4-diol (9d). *p*-Methoxypropiophenone (**1c**) (1.23 g, 7.5 mmol) was used as a ketone and $ZrCl_4$ (583 mg, 2.50 mmol) was chosen as a coordination metal. 4-Methoxybenzaldehyde (**3d**) (411 mg, 2.5 mmol) was added. The crude product was recrystallized from ethanol furnishing 374 mg (800 µmol, 32%) of **9d**. ^{1}H NMR (400 MHz, DMSO-d$_6$): δ 0.27 (d, $J = 6.8$ Hz, 3H), 0.37 (d, $J = 6.8$ Hz, 3H), 2.24−2.32 (m, 2H), 3.72 (s, 3H), 3.73 (s, 3H), 3.74 (s, 3H), 4.83 (d, $J = 10.4$ Hz, 1H), 5.33 (s, 1H), 6.86 (d, $J = 9.2$ Hz, 2H), 6.89 (d, $J = 10.6$ Hz, 2H), 6.92 (d, $J = 8.4$ Hz, 2H), 7.22 (s, 1H), 7.32 (br. d, $J = 8.0$ Hz, 1H), 7.45 (d, $J = 8.4$ Hz, 2H), 7.51−7.55 ppm (m, 3H). ^{13}C NMR (100 MHz, DMSO-d$_6$): δ 9.7, 10.8, 46.0, 47.1, 54.8, 54.9, 55.0, 74.3, 77.3, 100.6, 112.2, 112.6, 113.3, 113.9, 125.2, 127.5, 128.9, 133.6, 136.5, 137.0, 157.4, 158.6 ppm. IR (KBr): 3425, 2978, 2939, 2907, 1611, 1511, 1467, 1385, 1299, 1243, 1102, 1060, 1035, 966, 823, 549 cm^{-1}. Anal. Calcd for $C_{28}H_{32}O_6$ (464.55 g mol^{-1}): C 72.39, H 6.94; Found: C 72.75, H 6.63.

X-ray data of 5a and 7h

Table S1. Crystal data and structure refinement for **5a**.

Name	**5a**
Empirical Formula	$C_{25} H_{26} O_3$
Formula weight [g·mol^{-1}]	374.46
Temperature [K]	120(2)
Wavelength [pm]	71.073
Instrument, scan-type	STOE-IPDS, ϕ-Scan, Δϕ = 1.5°, 137
Crystal system, space group	orthorhombic, $P2_12_12_1$, *o*P216
Unit cell dimensions [pm]	a = 872.2(2)
	b = 1295.3(3)
	c = 1754.7(4)
Volume [nm^3]	1.9824(8)
Density [Mg·m^{-3}]	4, 1.255
Absorption coefficient [mm^{-1}]	0.081
F(000)	800
Crystal size [mm^3]	0.55 × 0.5 × 0.45
∅ range for the data collection [°]	2.82 − 25.05
Index ranges (H)	−10 ≤ h ≤ 10, −15 ≤ k ≤ 15, −20 ≤ l ≤ 20
Reflections collected / unique	14713 / 3513 / 3262
$R_{int.}$, $R_σ$	0.0969, 0.0575
$T_{Min.}$, $T_{Max.}$	0.9381, 0.9683
Refinement method	Least-squares (Full Matrix) on F^2
Data / restraints / Parameters	3513 / 0 / 277
S(F^2)	1.062
R indices (I > 2 σ (I))	R1 = 0.0333, wR2 = 0.0815
R indices (total)	R1 = 0.0365, wR2 = 0.0830
$Δρ_{min.}$, $Δρ_{max.}$ [10^{-6}e·pm^{-3}]	−0.20(1), 0.14(1)

Table S2. Crystal data and structure refinement for **7h**.

Name	**7h**
Empirical Formula	$C_{19} H_{26} O_3$
Formula weight [g·mol^{-1}]	302.40
Temperature [K]	173(1)
Wavelength [pm]	71.073 (Mo-Kα)
Instrument, scan-type	STOE-IPDS, ϕ-Scan = 1.5°
Crystal system, space group	$P2_1/c$ (Nr.: 14)
Unit cell dimensions [pm]	a = 996.9(2)
	b = 1840.6(4), β = 109.37(3)
	c = 907.0(2)
Volume [nm^3]	1.5701(6)
Density [Mg·m^{-3}]	4, 1.279
Absorption coefficient [mm^{-1}]	0.085
F(000)	656
Crystal size [mm^3]	1.8 × 0.7 × 0.7
∅ range for the data collection [°]	2.63 − 28.18
Index ranges (H)	−13 ≤ h ≤ 13, −24 ≤ k ≤ 24, −11 ≤ l ≤ 11
Reflections collected / unique	12197 / 3572 / 3027
$R_{int.}$, R_σ	0.1523, 0.0863
$T_{Min.}$, $T_{Max.}$	Not applicable (μ·x ≤ 0.1)
Refinement method	Least-squares (Full Matrix) on F^2
Data / restraints / Parameters	3572 / 0 / 204
$S(F^2)$	1.051
R indices (I > 2 σ (I))	R1 = 0.0615, wR2 = 0.1808
R indices (total)	R1 = 0.0689, wR2 = 0.1891
$\Delta\rho_{min.}$, $\Delta\rho_{max.}$ [10^{-6}e·pm^{-3}]	−0.408, 0.771

^{1}H and ^{13}C NMR Spectra

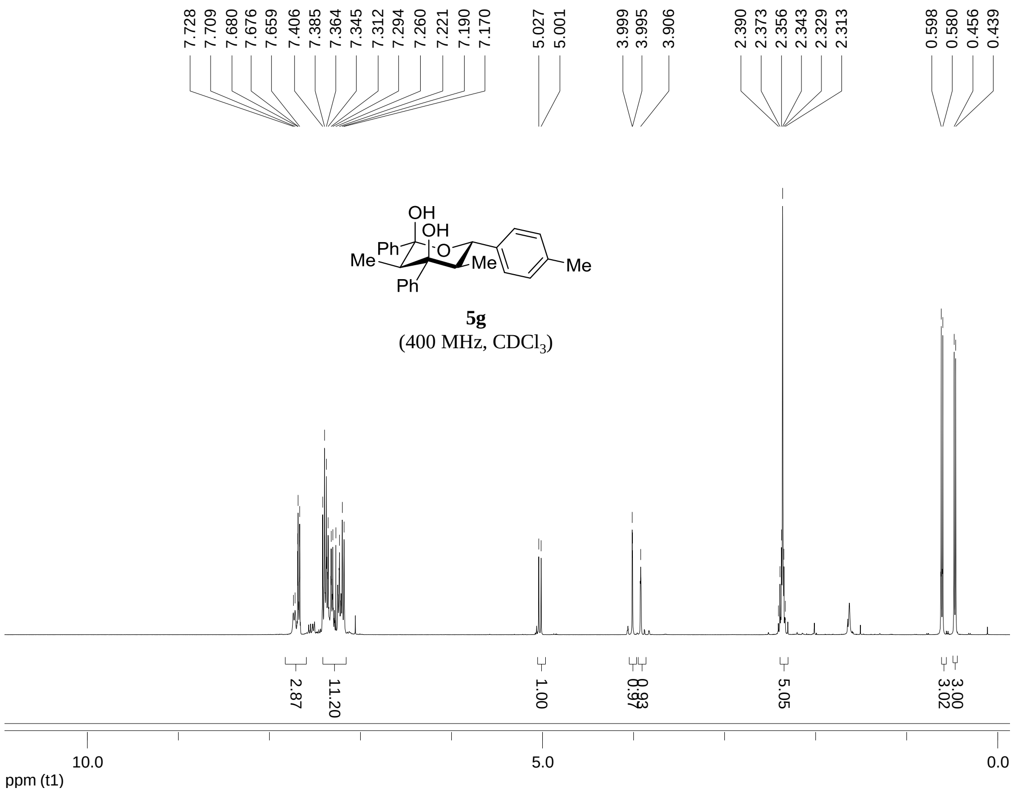

Figure S1. ^{1}H NMR (400 MHz) of compound **5g**.

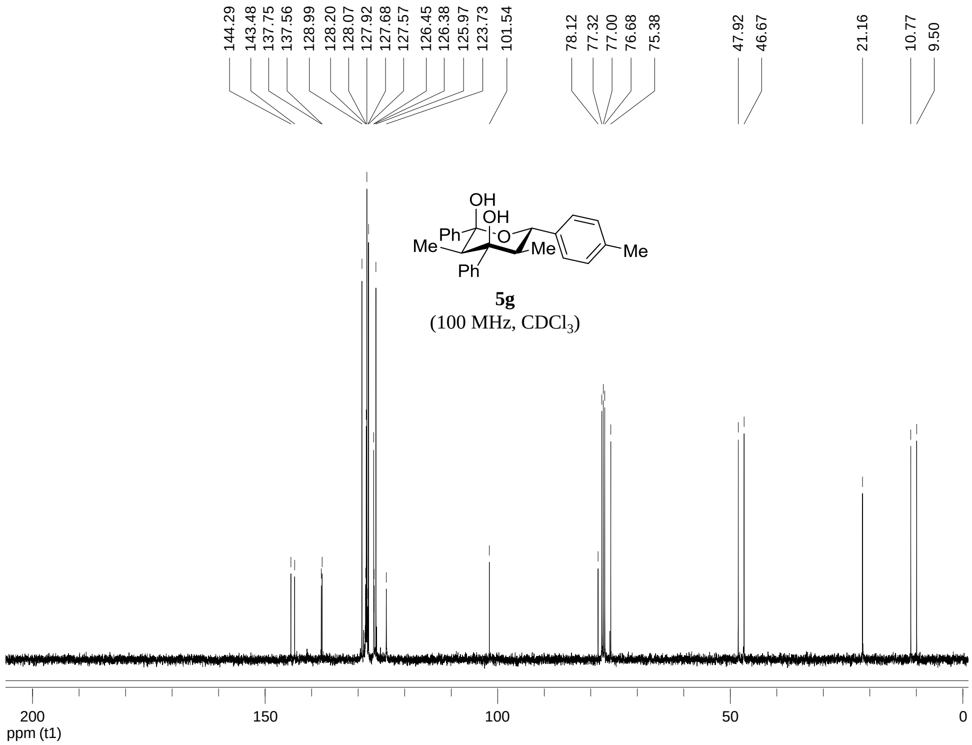

Figure S2. ^{13}C NMR (100 MHz) of compound **5g**.

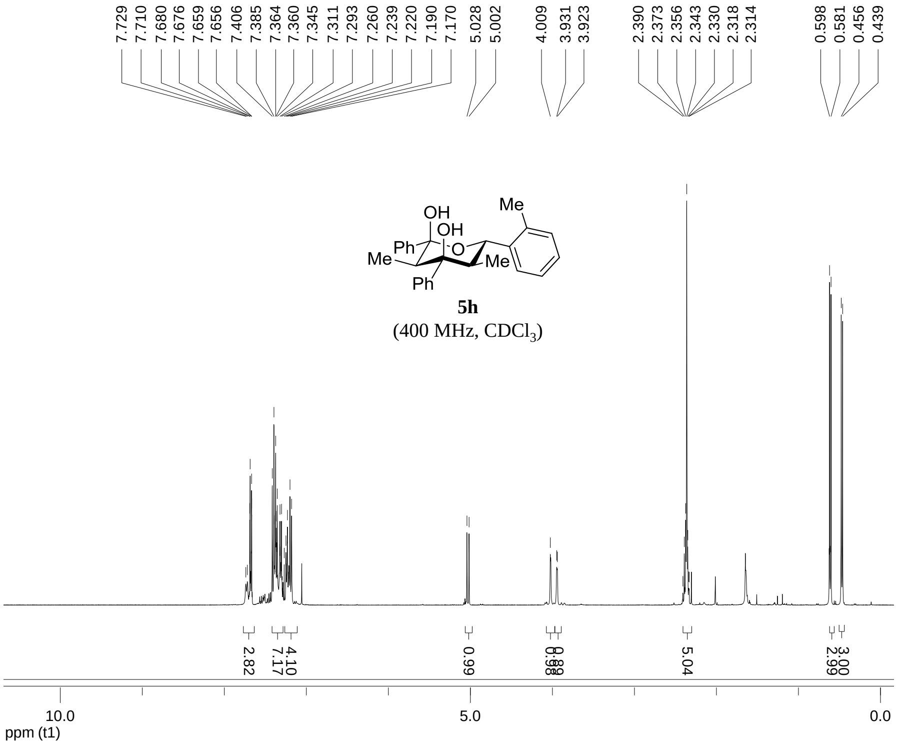

Figure S3. ^{1}H NMR (400 MHz) of compound **5h**.

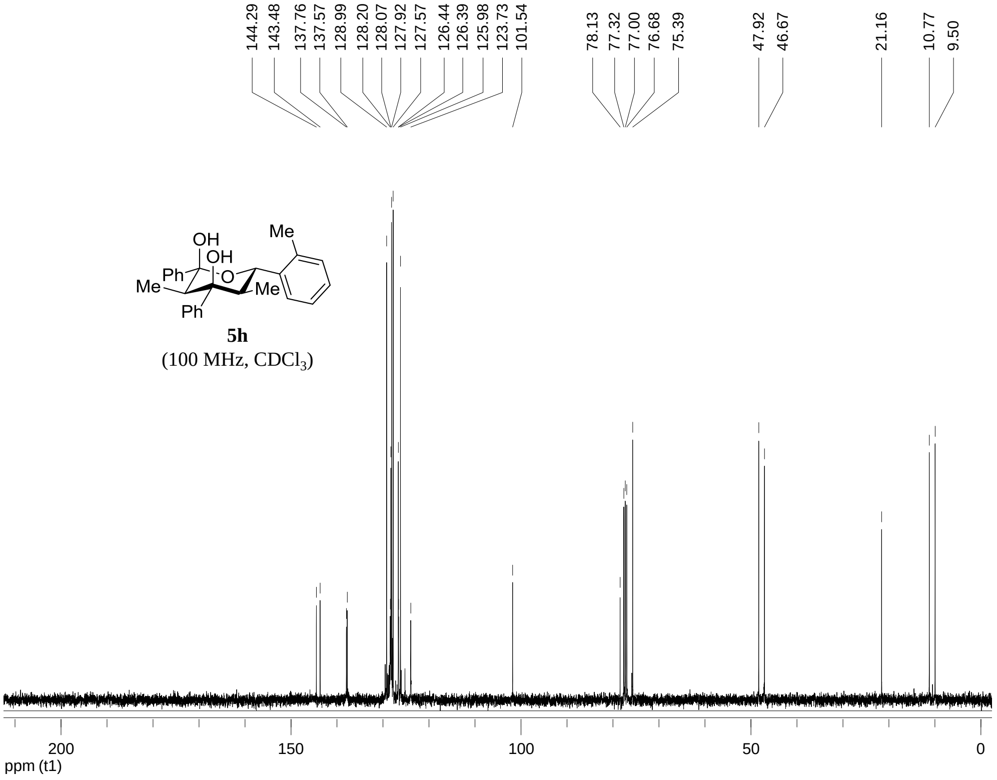

Figure S4. ^{13}C NMR (100 MHz) of compound **5h**.

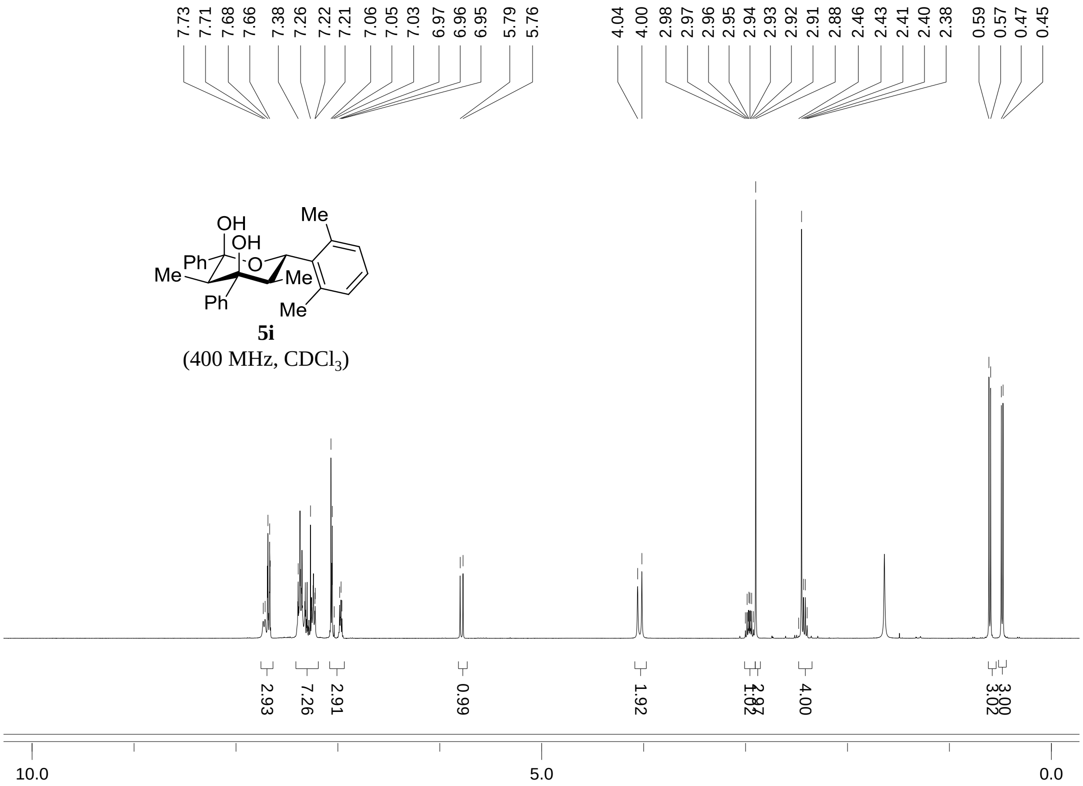

Figure S5. ^{1}H NMR (400 MHz) of compound **5i**.

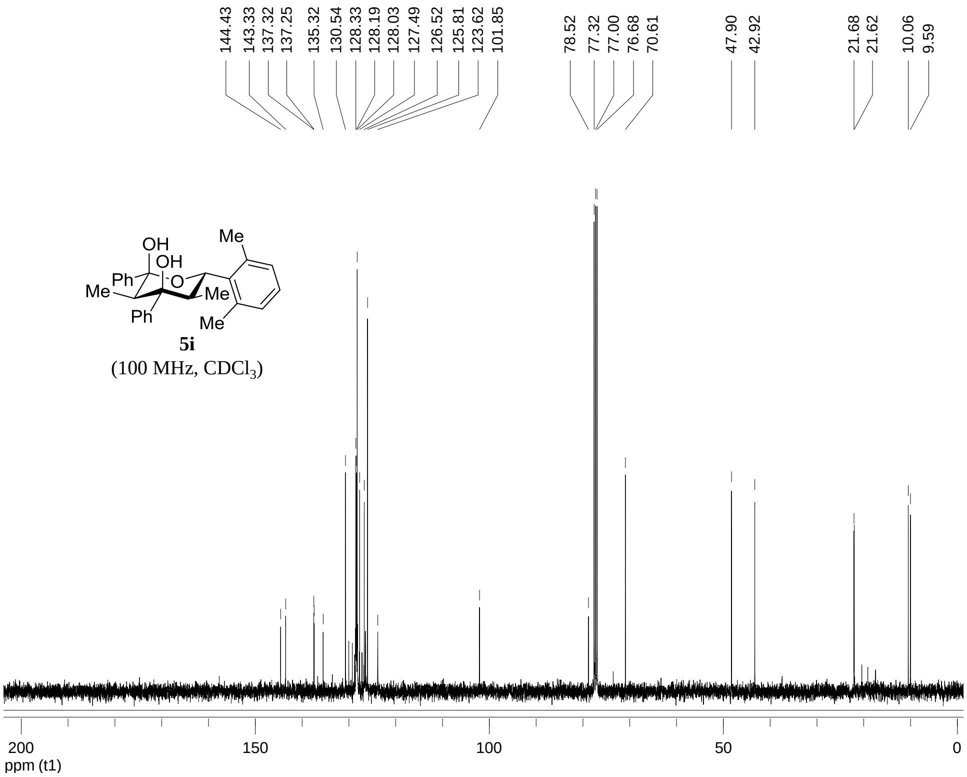

Figure S6. ^{13}C NMR (100 MHz) of compound **5i**.

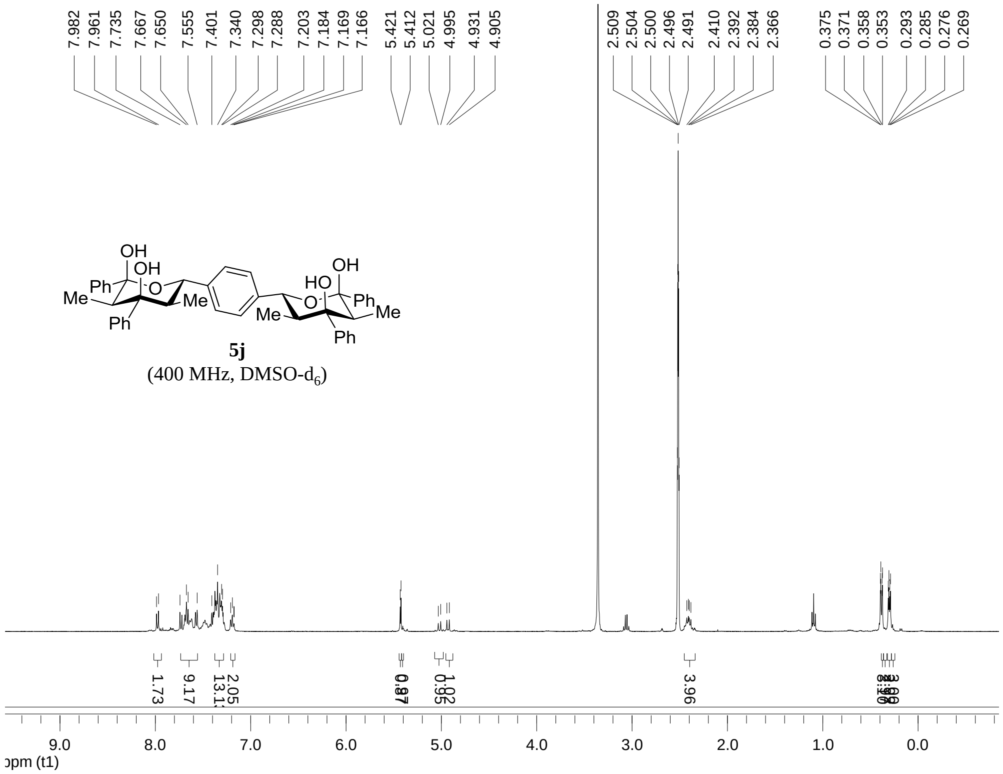

Figure S7. ^{1}H NMR (400 MHz) of compound **5j**.

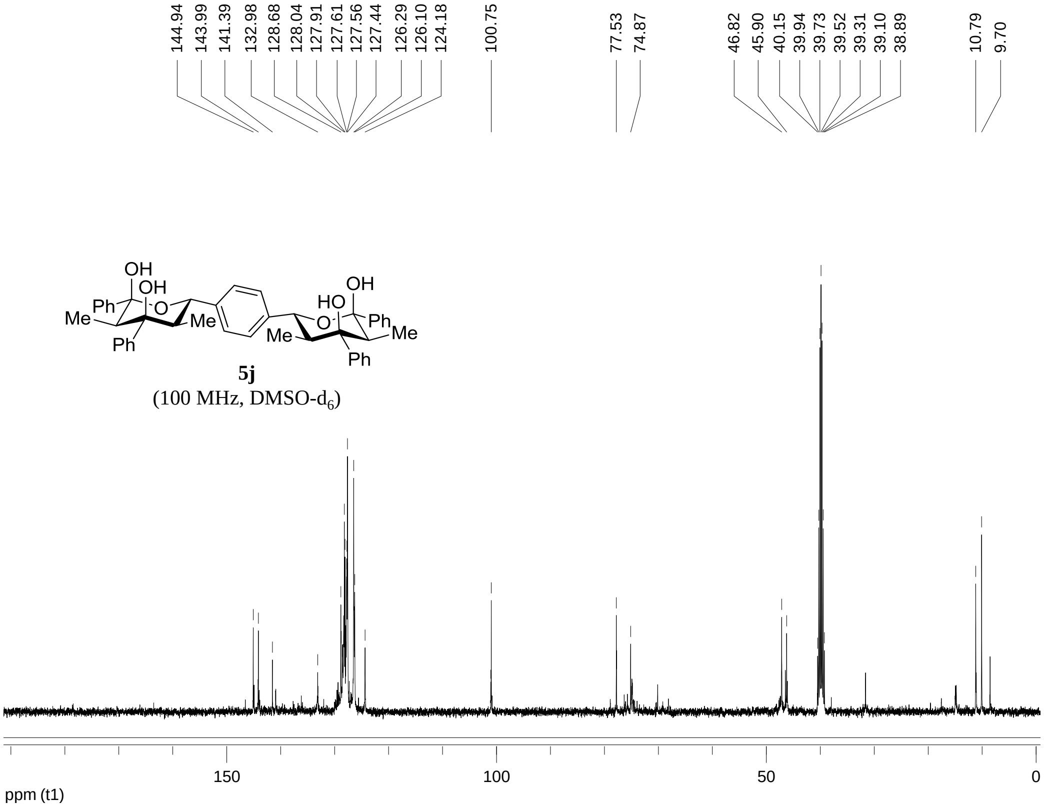

Figure S8. ^{13}C NMR (100 MHz) of compound **5j**.

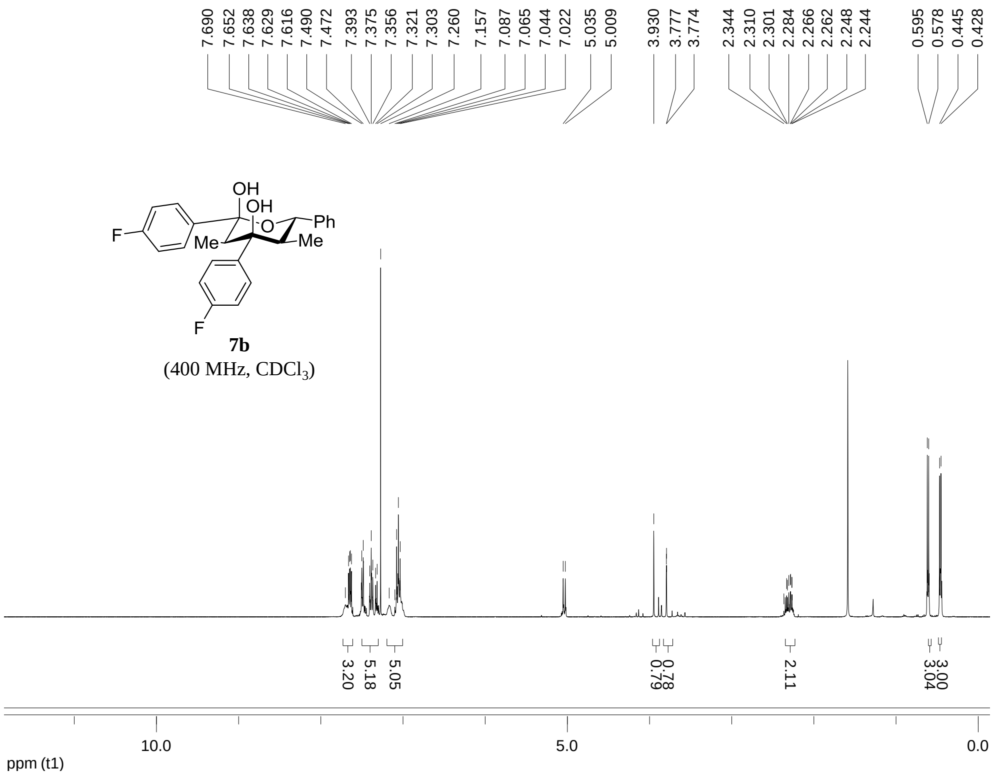

Figure S9. ^{1}H NMR (400 MHz) of compound **7b**.

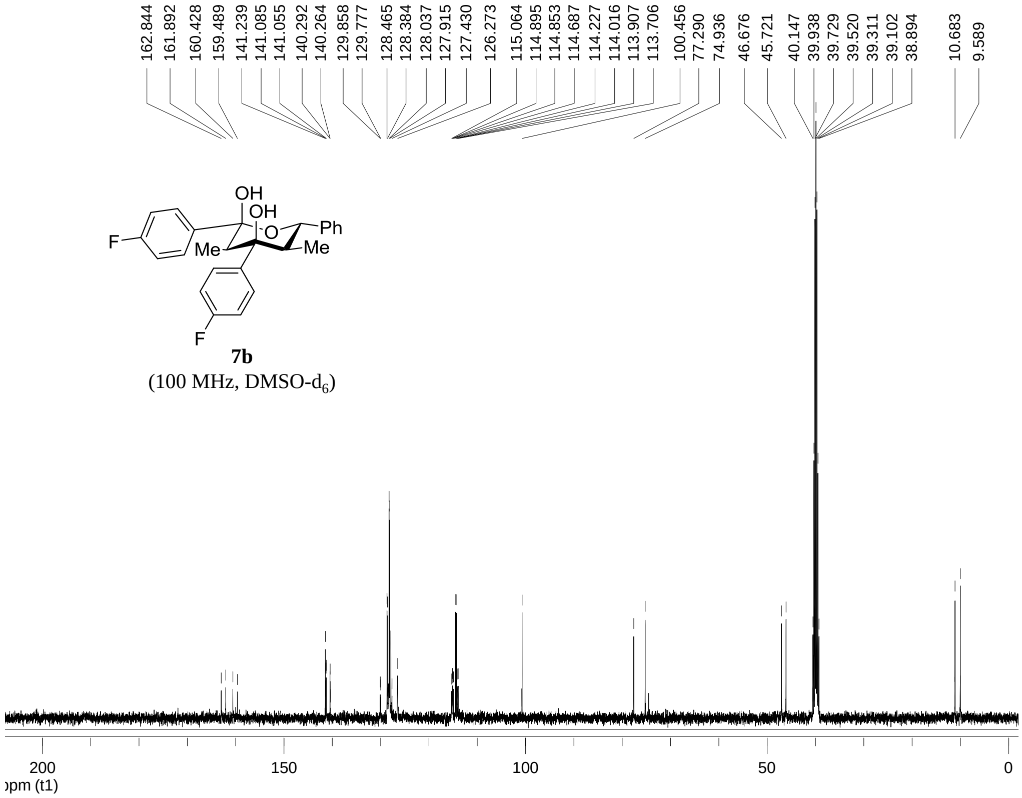

Figure S10. ^{13}C NMR (100 MHz) of compound **7b**.

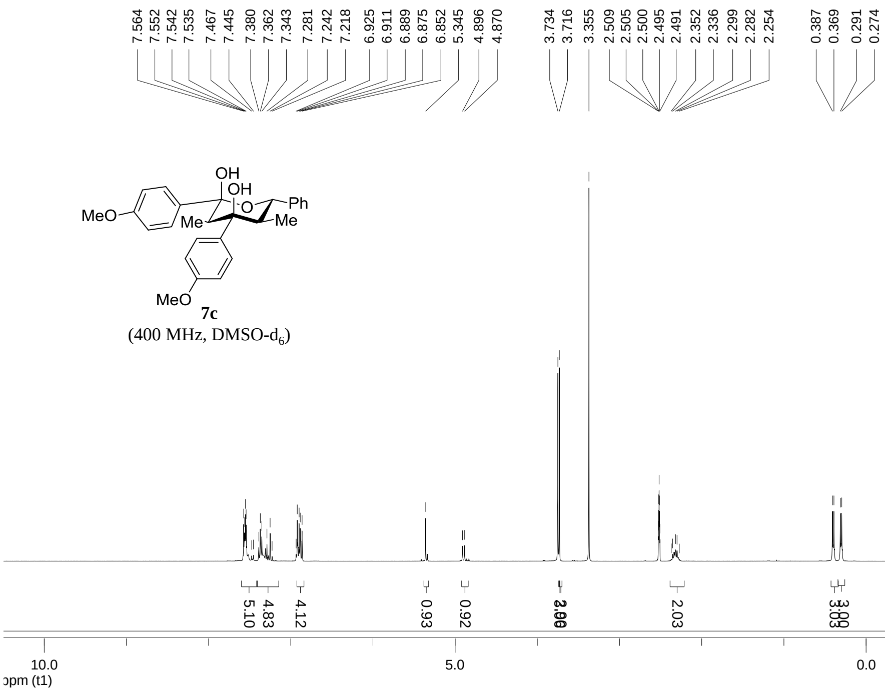

Figure S11. ^{1}H NMR (400 MHz) of compound **7c**.

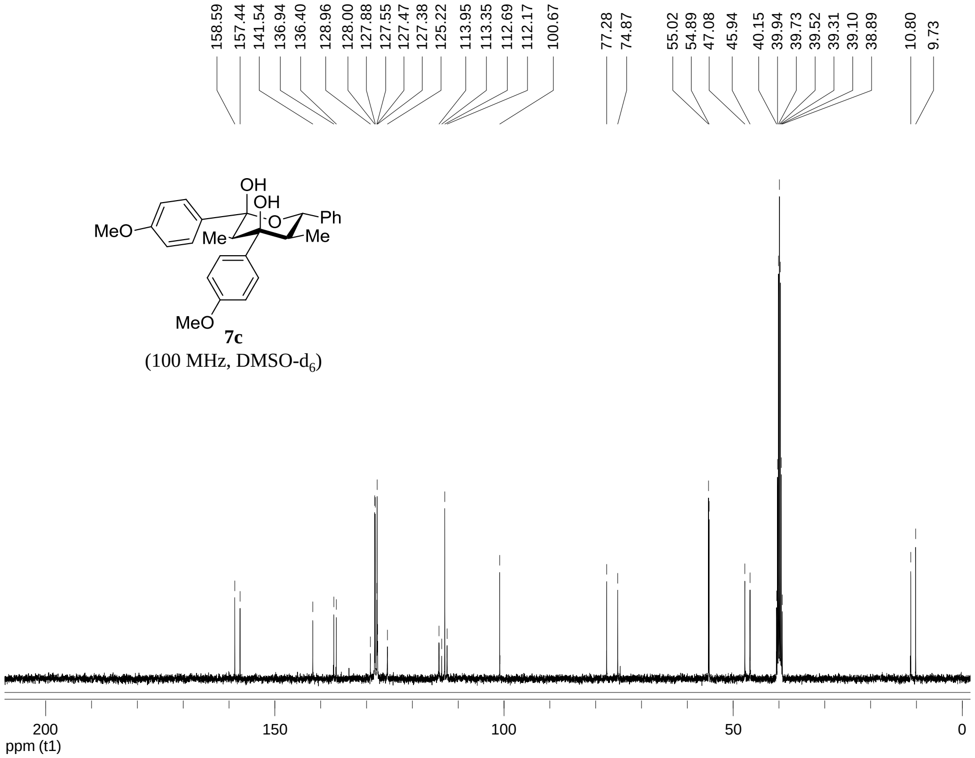

Figure S12. ^{13}C NMR (100 MHz) of compound **7c**.

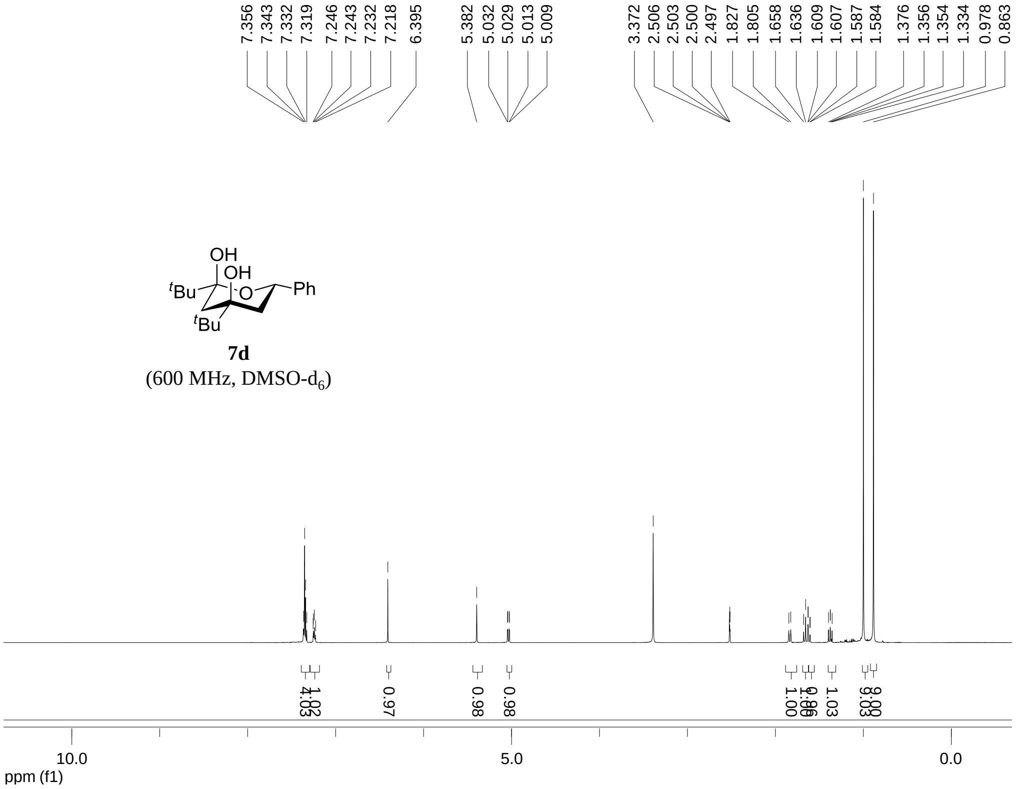

Figure S13. ^{1}H NMR (600 MHz) of compound **7d**.

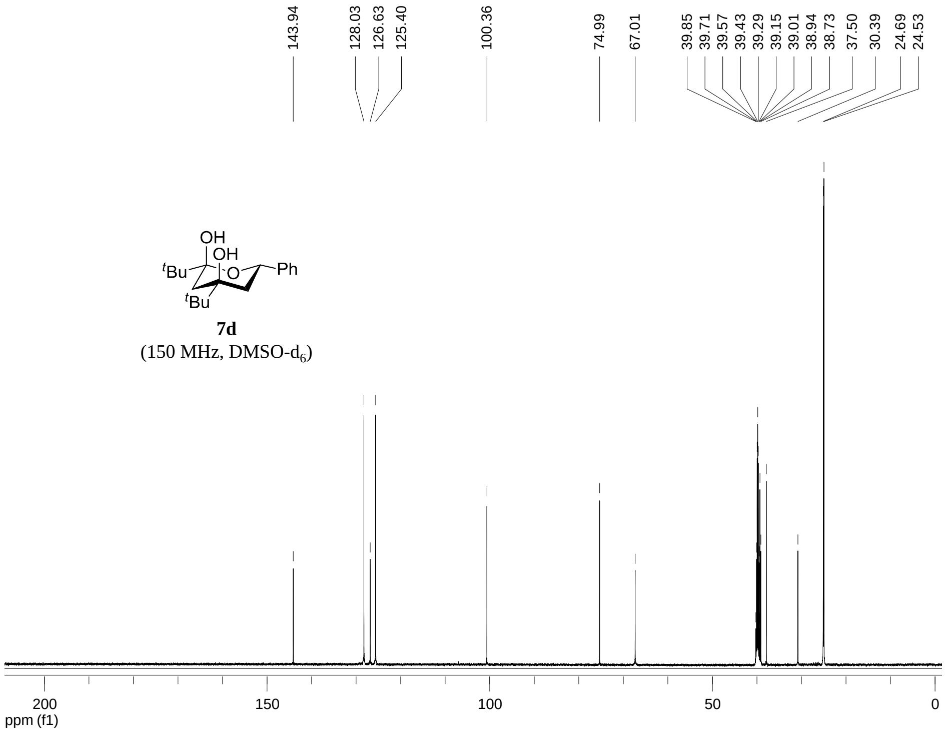

Figure S14. ^{13}C NMR (150 MHz) of compound **7d**.

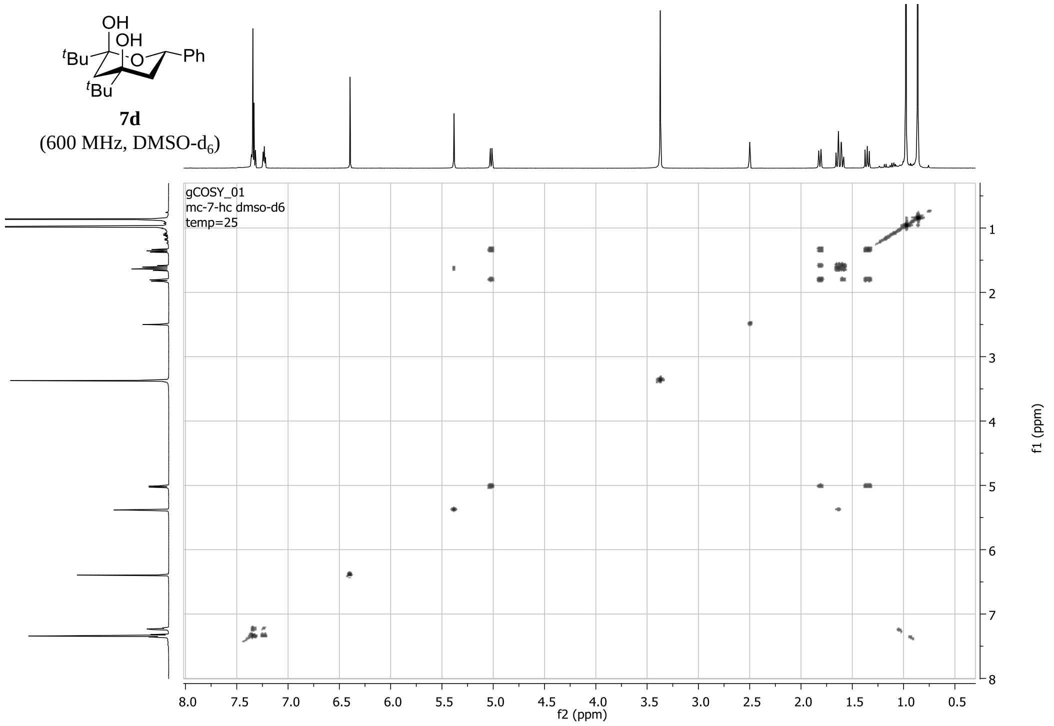

Figure S15. $^{1}H,^{1}H$-COSY NMR (600 MHz) of compound **7d**.

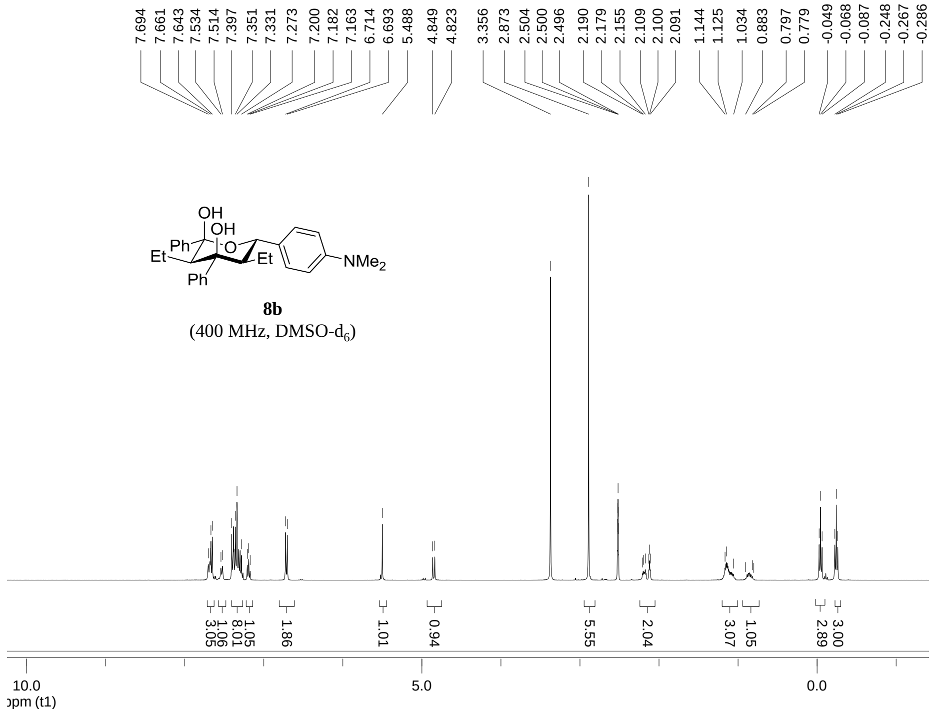

Figure S16. ^{1}H NMR (400 MHz) of compound **8b**.

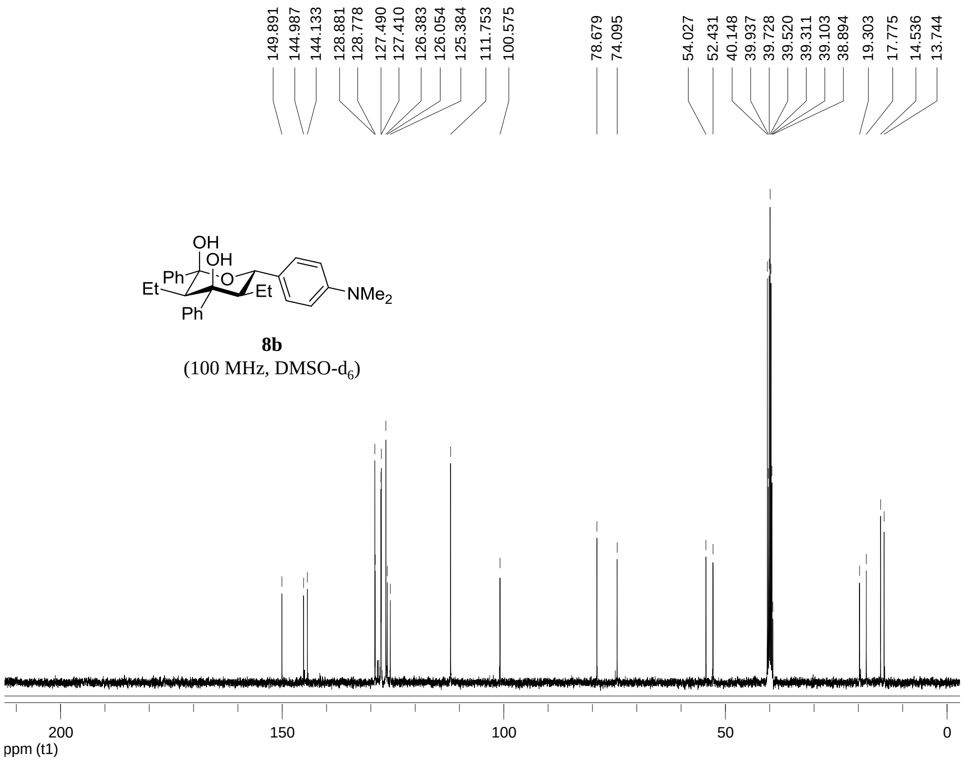

Figure S17. ^{13}C NMR (100 MHz) of compound **8b**.

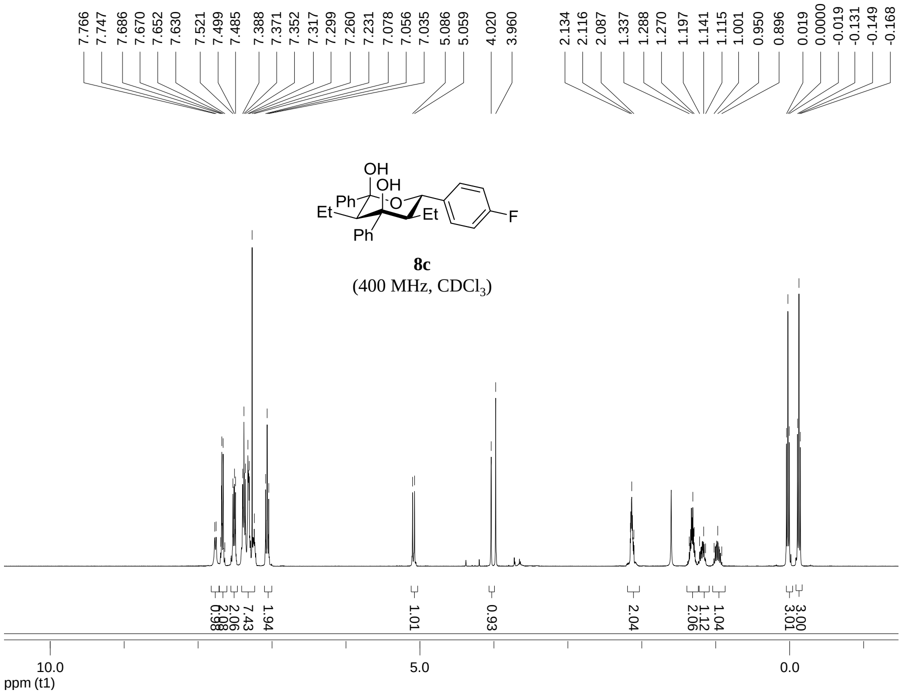

Figure S18. ^{1}H NMR (400 MHz) of compound **8c**.

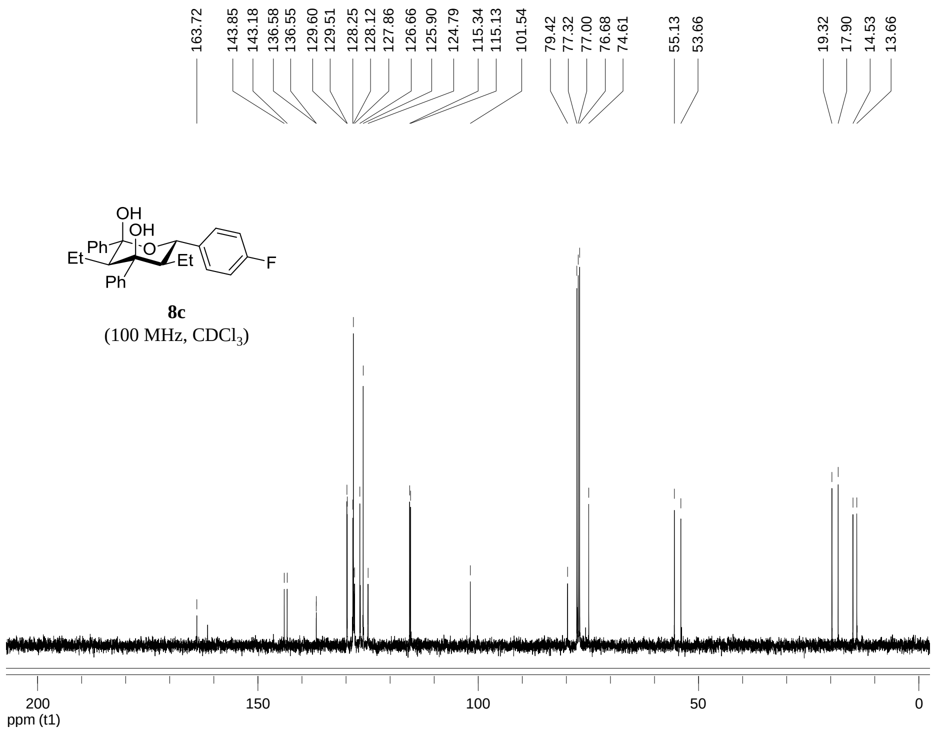

Figure S19. ^{13}C NMR (100 MHz) of compound **8c**.

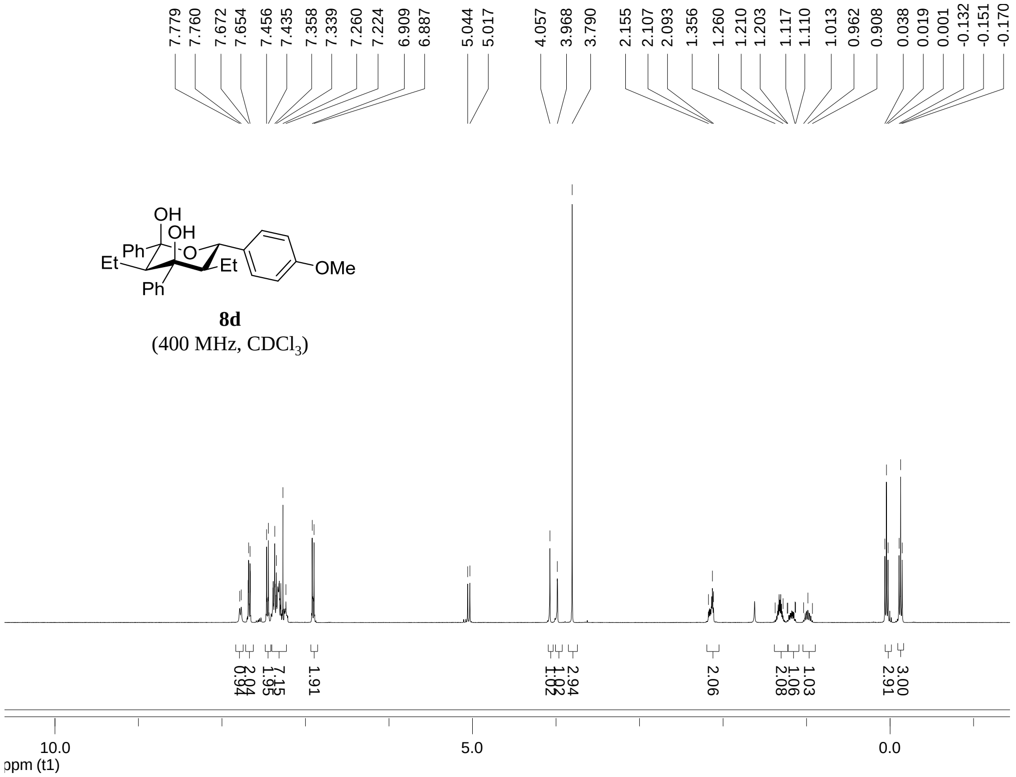

Figure S20. ^{1}H NMR (400 MHz) of compound **8d**.

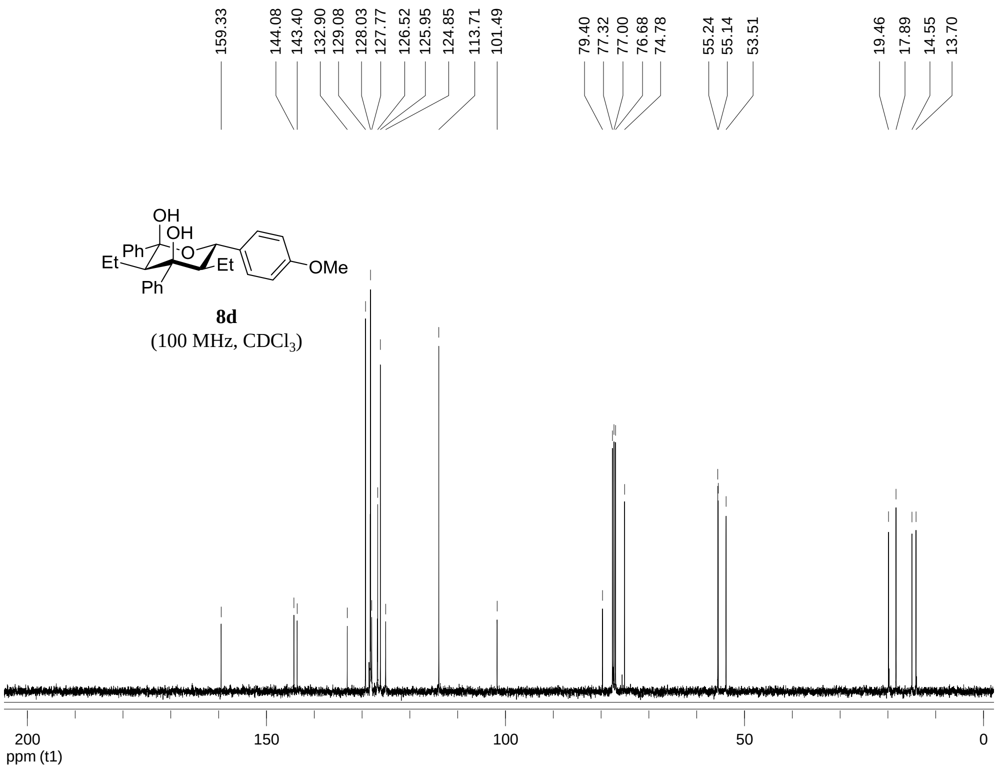

Figure S21. ^{13}C NMR (100 MHz) of compound **8d**.

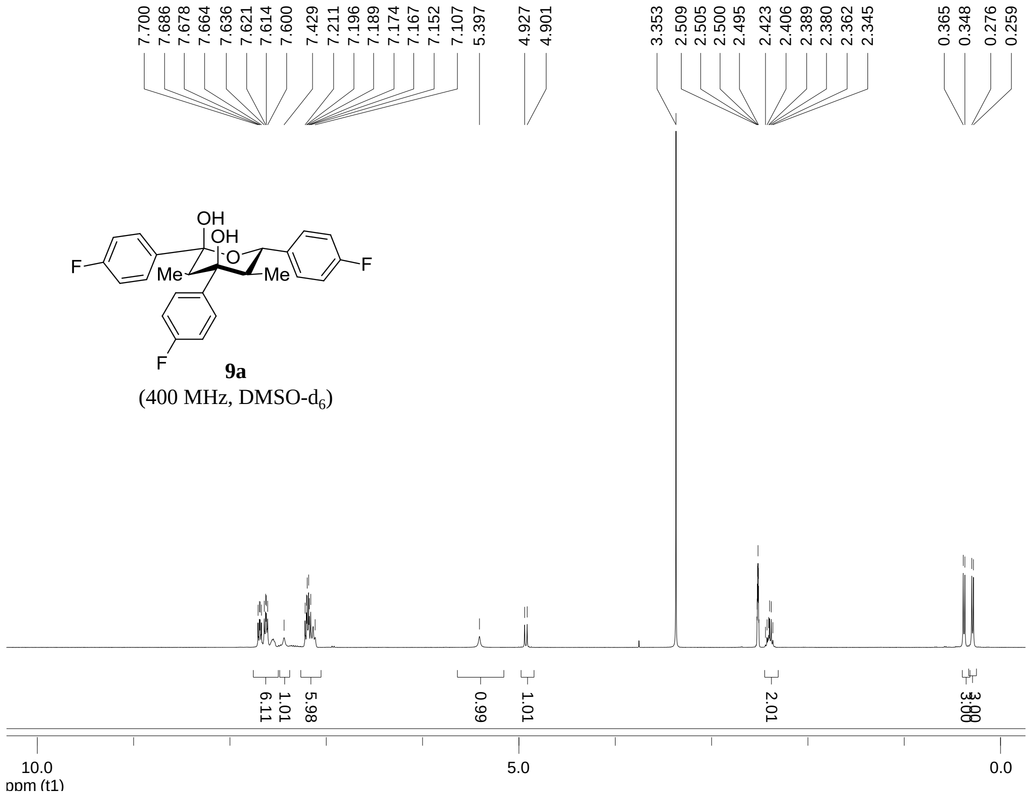

Figure S22. ^{1}H NMR (400 MHz) of compound **9a**.

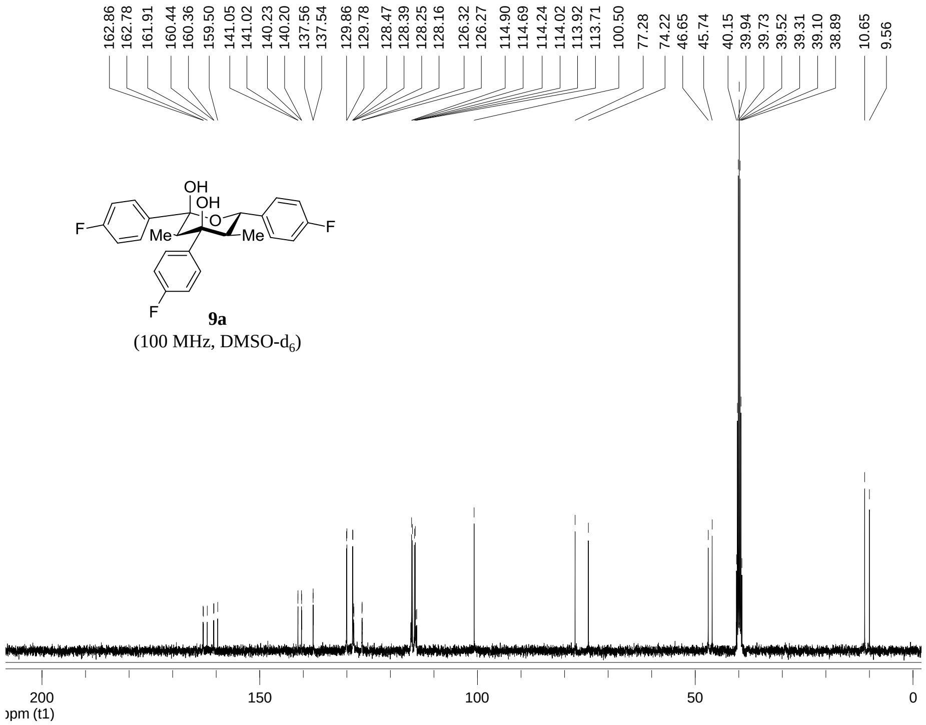

Figure S23. ^{13}C NMR (100 MHz) of compound **9a**.

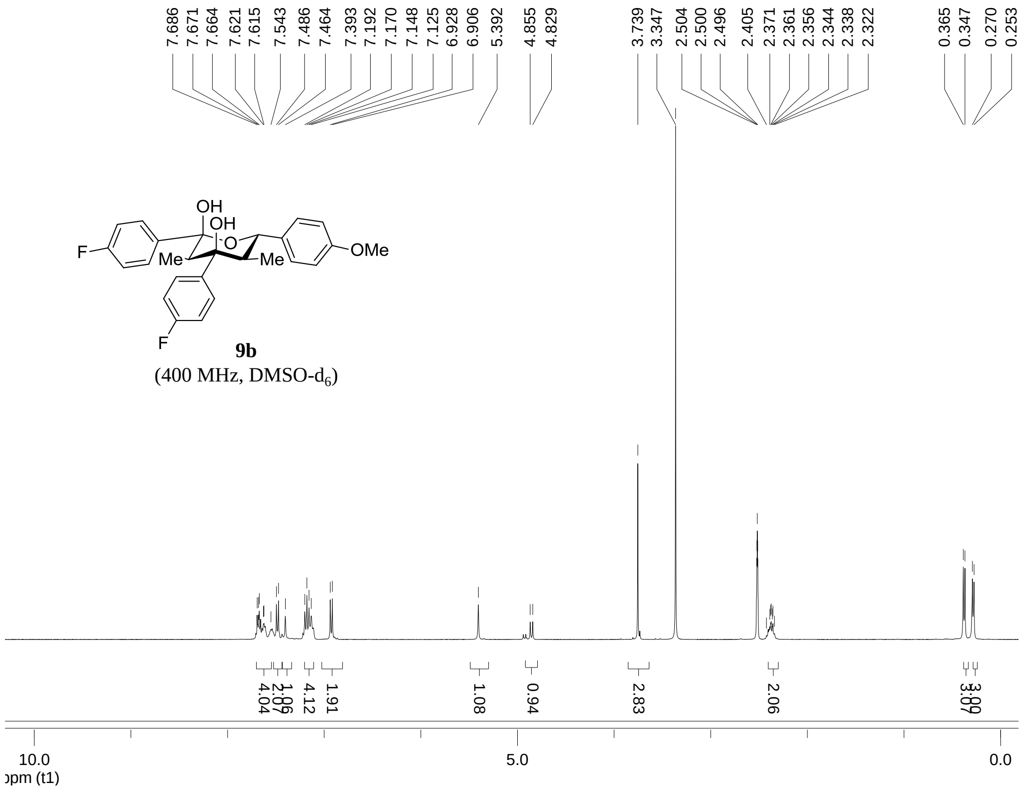

Figure S24. ^{1}H NMR (400 MHz) of compound **9b**.

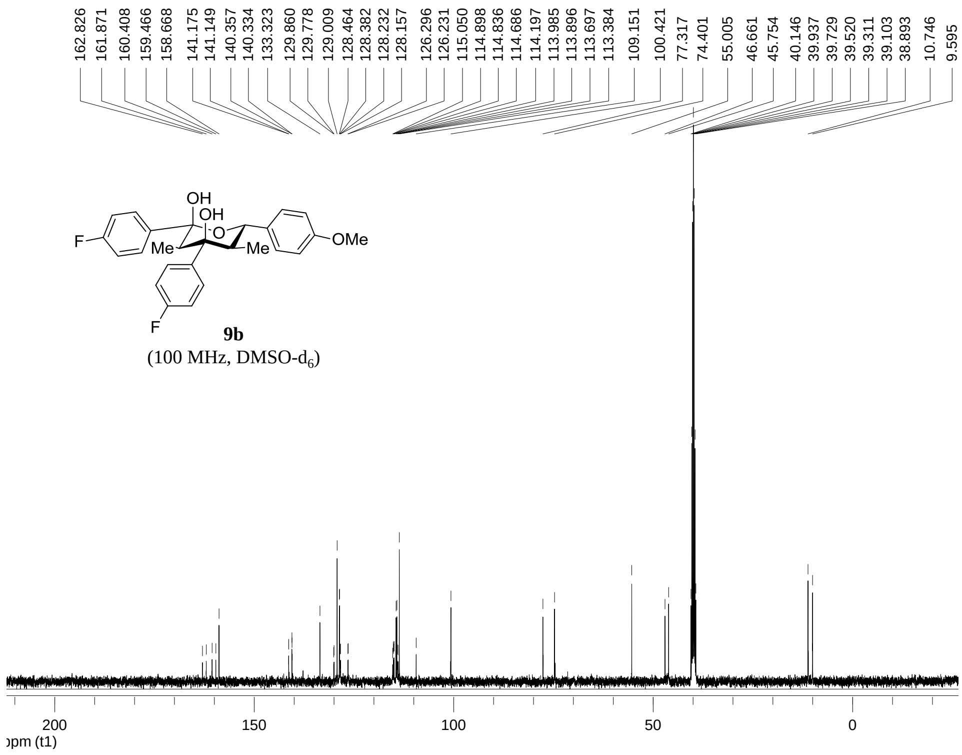

Figure S25. ^{13}C NMR (100 MHz) of compound **9b**.

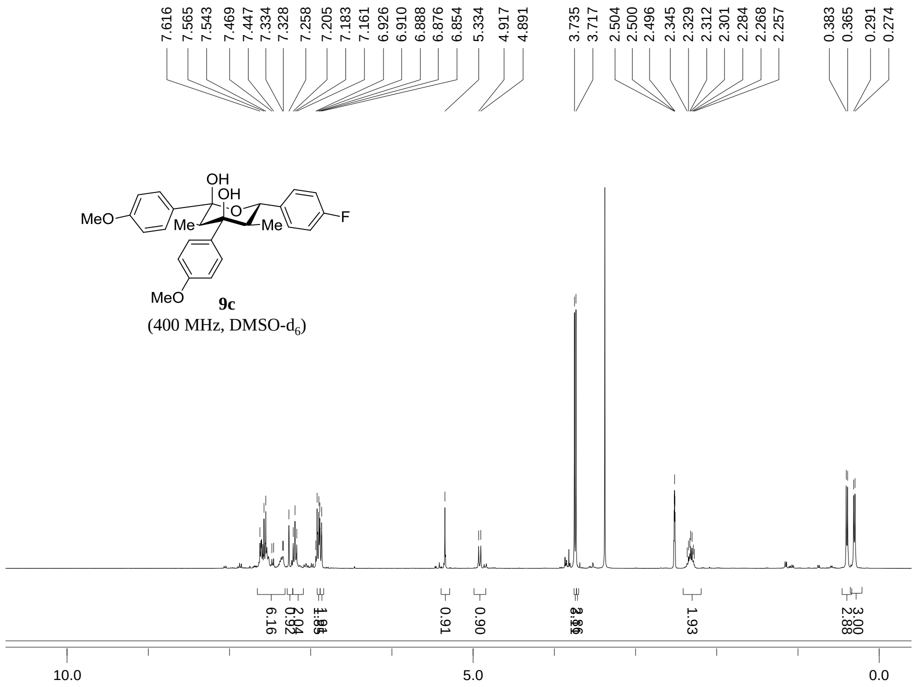

Figure S26. ^{1}H NMR (400 MHz) of compound **9c**.

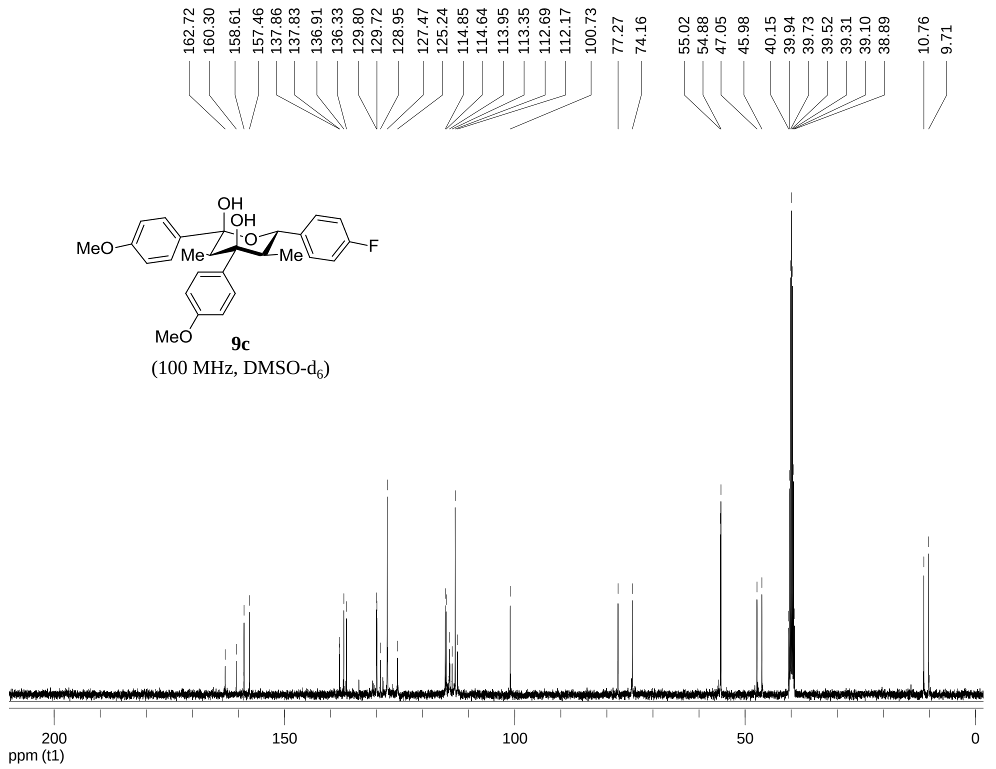

Figure S27. ^{13}C NMR (100 MHz) of compound **9c**.

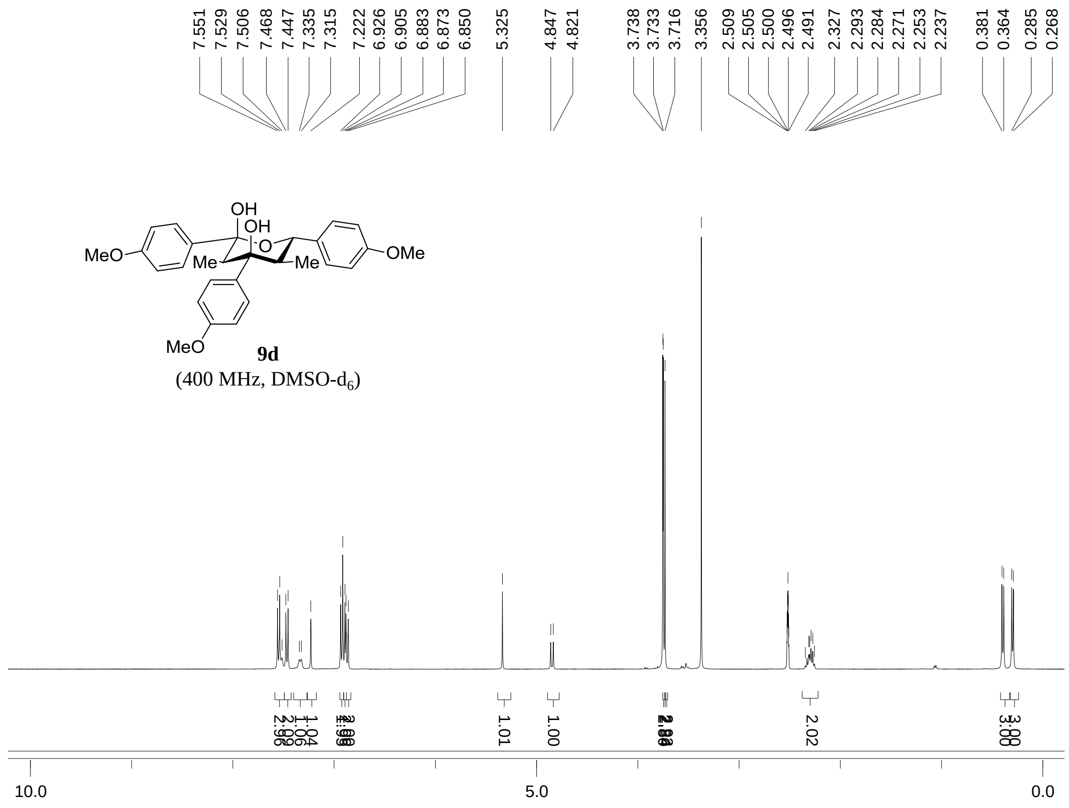

Figure S28. ^{1}H NMR (400 MHz) of compound **9d**.

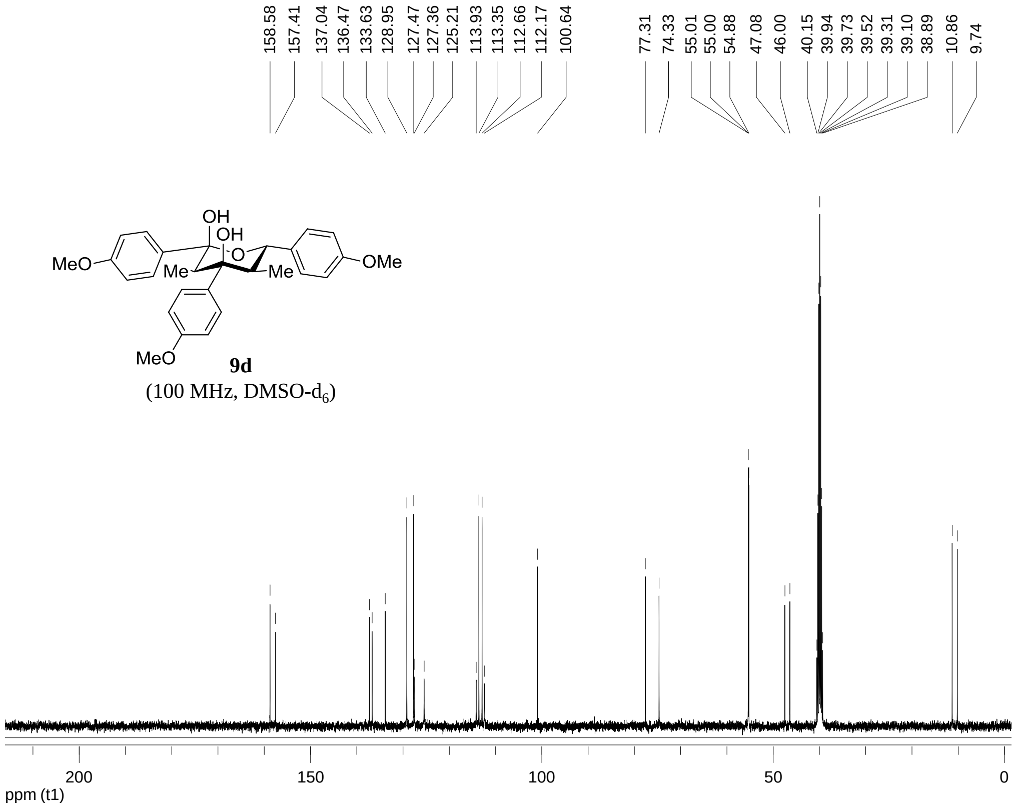

Figure S29. ^{13}C NMR (100 MHz) of compound **9d**.

Cartesian Coordinates

Complex (C)

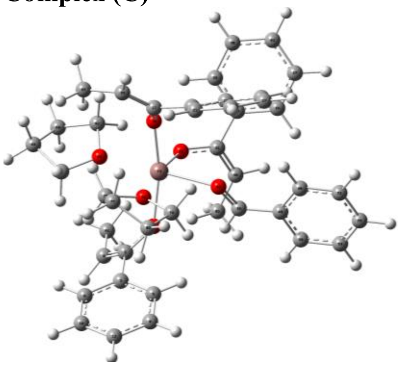

Energy: -2083.22612943 a.u.
O 1.6007860 0.9715610 -1.1898660
C 2.8912560 1.2312750 -1.4456070
C 3.5864530 2.1034300 -0.4461250
C 3.5393500 0.8541760 -2.5763570
C 2.8770680 3.1351590 0.1913280
C 4.9460700 1.9449790 -0.1266130
H 4.5533070 1.2203560 -2.7186220
C 3.5056730 3.9914810 1.0951500
H 1.8271510 3.2658460 -0.0500650
C 5.5761600 2.7947630 0.7844290
H 5.5091390 1.1405610 -0.5919370
C 4.8602360 3.8242440 1.3993780
H 2.9399700 4.7962170 1.5593280
H 6.6278990 2.6480910 1.0184520
H 5.3506270 4.4880370 2.1066420
O -0.1933350 -2.0029230 0.7197320
C -0.3665280 -2.2798850 2.0204670
C -1.4875510 -1.5771760 2.7216240
C 0.3397070 -3.2400980 2.6701280
C -2.6388400 -1.2156770 2.0024850
C -1.4523030 -1.2921410 4.0988850
H 0.0494900 -3.4857930 3.6879440
C -3.7277580 -0.6233970 2.6440940
H -2.6824320 -1.4291720 0.9411050
C -2.5350900 -0.6875510 4.7382350
H -0.5630070 -1.5412790 4.6716350
C -3.6822780 -0.3533540 4.0143640
H -4.6190480 -0.3863820 2.0676310
H -2.4808210 -0.4742430 5.8032580
H -4.5294190 0.1108820 4.5131770
O -0.5989600 -0.8717310 -2.0685880
C -1.7691100 -0.3689720 -2.4611730
C -2.9938990 -1.0763000 -1.9716320
C -4.2446190 -0.4415680 -1.8613520
C -2.9063690 -2.4312900 -1.6097740
C -1.2450260 2.0033510 -0.6596370
C -5.3697330 -1.1438690 -1.4305190
H -4.3336810 0.6152470 -2.0939150
C -4.0329370 -3.1355180 -1.1852200
H -1.9400890 -2.9195030 -1.6565860
H -0.5241870 2.2689040 -1.4407420
C -5.2716150 -2.4971510 -1.0959130
H -6.3248770 -0.6303380 -1.3492920
H -3.9407500 -4.1846820 -0.9157280
H -6.1484860 -3.0439720 -0.7590120
C -1.8740820 0.6919800 -3.3133150
O -0.9831960 1.0625160 0.1173670
C -2.4193140 2.8567030 -0.4957030
C -3.3712560 2.5955320 0.5070400
C -2.5741780 3.9726410 -1.3366220
C -4.4642830 3.4431740 0.6559190
H -3.2364290 1.7322520 1.1508530
C -3.6687880 4.8180610 -1.1811660
H -1.8344640 4.1664730 -2.1093820
C -4.6140950 4.5522440 -0.1851380
H -5.2023440 3.2440360 1.4277840
H -3.7885060 5.6799600 -1.8311570
H -5.4694930 5.2114110 -0.0630120
H -2.8669980 0.9630120 -3.6611190
O 1.9731700 -2.0716180 -1.1838130
C 3.3935590 -2.2825470 -0.9446260
C 1.4124560 -3.1842190 -1.9455110
C 3.7150710 -3.6530410 -1.5511470
H 3.9277340 -1.4607150 -1.4278150
H 3.5743810 -2.2465480 0.1330280
C 2.6206860 -3.8252030 -2.6173580
H 0.9151580 -3.8616920 -1.2432750
H 0.6745160 -2.7560320 -2.6231280
H 3.6302580 -4.4385090 -0.7916580
H 4.7280110 -3.6886710 -1.9630030
H 2.4412780 -4.8721770 -2.8796760
H 2.8818030 -3.2795390 -3.5306260
In 0.4920160 -0.5006960 -0.3930800
O 1.5226420 0.1804500 1.6283580
C 2.6603020 -0.4707400 2.2468710
C 0.9976140 1.2254290 2.4972550
C 2.6192270 -0.0444970 3.7119540
H 2.5403440 -1.5468220 2.1108360
H 3.5704830 -0.1217440 1.7441920
C 2.0249530 1.3690960 3.6201110
H 0.8819600 2.1298770 1.8992380
H 0.0209620 0.8982410 2.8644890
H 3.6085950 -0.0679670 4.1786320
H 1.9533240 -0.7097410 4.2720040
H 2.7978260 2.0918540 3.3371130
H 1.5635040 1.7019300 4.5548070
C 1.4184540 -4.0789960 2.0501630
H 1.1112070 -5.1316450 1.9537800
H 1.6607040 -3.7131440 1.0489500
H 2.3400530 -4.0842030 2.6523970
C 2.9421740 0.0538670 -3.6972030
H 1.9577050 -0.3360420 -3.4292520
H 2.8250310 0.6619060 -4.6067840
H 3.5860500 -0.7931480 -3.9796330
C -0.7168070 1.4034370 -3.9505380
H -0.5556970 1.0628240 -4.9850790
H -0.8963440 2.4870320 -4.0124810
H 0.2109010 1.2358310 -3.3986790

TS-C-A1

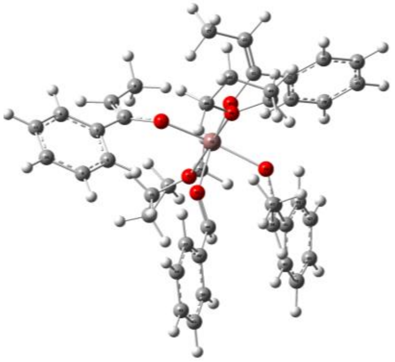

Energy: -2083.22729589 a.u.
Frequency: -172.3947 cm^{-1}
O -1.2879650 -1.9030810 0.4208660
C -2.0030720 -2.4848590 1.3925270
C -3.4867970 -2.4047340 1.1917740
C -1.4920460 -3.1173460 2.4798400
C -4.0365910 -1.3188770 0.4889040
C -4.3658220 -3.3924050 1.6703830
H -2.2031300 -3.5217550 3.1954420
C -0.0396720 -3.3340600 2.7914750
C -5.4148940 -1.2067410 0.3037390
H -3.3665370 -0.5593720 0.1012360
C -5.7430800 -3.2829880 1.4816310
H -3.9629180 -4.2666810 2.1729720
H 0.2393290 -2.8964490 3.7638900
H 0.1922800 -4.4069850 2.8697520
H 0.6170540 -2.9113630 2.0273470
C -6.2773460 -2.1865090 0.8001830
H -5.8168070 -0.3476870 -0.2293680
H -6.4002740 -4.0642590 1.8563450
H -7.3509110 -2.1029130 0.6504670
O 1.8158880 -1.5889240 0.4018730
C 2.6996830 -2.3090680 -0.3157290
C 4.0753630 -1.7236600 -0.3996000
C 2.4493860 -3.5267290 -0.8580190
C 4.5830690 -0.9650280 0.6688100
C 4.9047920 -1.9157850 -1.5180690
H 3.2851940 -4.0408340 -1.3262770
C 1.1523020 -4.2803970 -0.8002450
C 5.8770380 -0.4442110 0.6337870
H 3.9543780 -0.8060360 1.5377240
C 6.1960150 -1.3898540 -1.5586970
H 4.5250660 -2.4701340 -2.3714110
H 0.3510080 -3.6943560 -0.3448420
H 0.8211140 -4.5922180 -1.8032650
H 1.2582460 -5.2067970 -0.2161710
C 6.6919330 -0.6524610 -0.4813810
H 6.2539340 0.1201630 1.4841820
H 6.8131890 -1.5483130 -2.4398640
H 7.6978510 -0.2419570 -0.5125330
O 1.2365500 0.9669250 -0.9686370

C 1.0446400 2.2487870 -0.9650030
C 1.8961700 3.0607780 -0.0405110
C 1.5665590 4.3639720 0.3731400
C 3.1014720 2.4982760 0.4132540
C -1.4316380 2.1462180 -0.1584010
C 2.4139130 5.0792720 1.2180120
H 0.6393880 4.8242610 0.0451200
C 3.9551140 3.2213270 1.2460300
H 3.3613410 1.4950690 0.0933850
H -0.8549640 2.5924970 0.6619130
C 3.6142950 4.5127240 1.6547580
H 2.1373690 6.0817020 1.5343170
H 4.8911050 2.7736020 1.5695900
H 4.2784830 5.0754900 2.3053920
C 0.0614710 2.8456600 -1.7559740
O -1.4424730 0.8785580 -0.2880110
C -2.5795300 2.9581480 -0.6251180
C -3.5773730 2.3973860 -1.4377880
C -2.7055230 4.2932590 -0.2085600
C -4.6699180 3.1655740 -1.8372400
H -3.4877470 1.3584250 -1.7363430
C -3.7986400 5.0585500 -0.6072470
H -1.9441180 4.7265900 0.4367640
C -4.7828700 4.4966490 -1.4264360
H -5.4391240 2.7242510 -2.4653820
H -3.8877780 6.0898760 -0.2769620
H -5.6375790 5.0917920 -1.7361820
C -0.5316530 2.1628100 -2.9555800
H 0.1707100 2.1846300 -3.8015600
H -0.7611490 1.1162900 -2.7408580
H -1.4498030 2.6635820 -3.2787320
H 0.0094160 3.9301230 -1.7528470
In 0.1393400 -0.5884930 -0.0296480
O 0.2297390 0.2985770 2.0535090
C 1.4325780 0.2353350 2.8888860
C -0.9528240 0.4520300 2.8974820
C 0.9415710 0.3997600 4.3317250
H 1.9217560 -0.7180570 2.6909750
H 2.0814360 1.0536430 2.5669260
C -0.4163030 1.1016520 4.1656210
H -1.6713140 1.0549600 2.3427950
H -1.3809210 -0.5379200 3.0838790
H 1.6486380 0.9717430 4.9397740
H 0.8041840 -0.5797210 4.8026240
H -0.2806380 2.1797830 4.0189340
H -1.0839520 0.9516000 5.0192370
O -0.1741320 -1.3290650 -2.1221770
C 0.8233690 -1.3010760 -3.1873930
C -1.4025240 -1.9769100 -2.5765190
C 0.0348860 -1.6038810 -4.4577900
H 1.2936790 -0.3181510 -3.1592480
H 1.5696900 -2.0694830 -2.9640160
C -1.0552850 -2.5603130 -3.9453180
H -1.6889270 -2.7043530 -1.8194650
H -2.1770040 -1.2050070 -2.6382780
H 0.6639450 -2.0458660 -5.2362580
H -0.4147940 -0.6883730 -4.8599980
H -0.6523200 -3.5738300 -3.8376300
H -1.9288690 -2.6106180 -4.6022130

A1

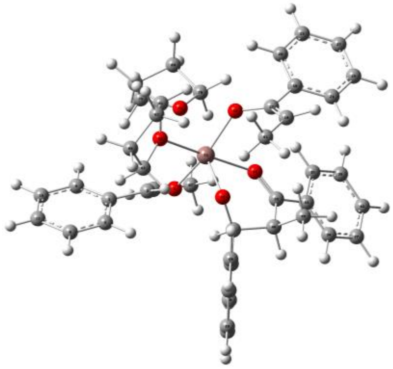

Energy: -2083.24307441 a.u.
O 1.4235640 -0.2668600 -1.2491190
C 2.0861710 -0.9050760 -2.2159210
C 3.5732660 -0.9763440 -2.0363180
C 1.5279860 -1.3638820 -3.3660060
C 4.2406950 0.0832690 -1.3973420
C 4.3438130 -2.0549100 -2.5039030
H 2.1962680 -1.7699550 -4.1210140
C 5.6280500 0.0739400 -1.2497060
H 3.6526950 0.9209420 -1.0359390
C 5.7308850 -2.0690990 -2.3497660
H 3.8474580 -2.8941480 -2.9837620
C 6.3812260 -1.0039510 -1.7231410
H 6.1240020 0.9142840 -0.7692680
H 6.3039540 -2.9184310 -2.7142540
H 7.4615400 -1.0141310 -1.6035780
O -0.9403230 -1.1378660 1.8256690
C -2.0808530 -0.6391990 2.3416290
C -3.3354790 -1.3151840 1.8848260
C -2.1458440 0.3211720 3.2944720
C -3.3670180 -2.7135870 1.7449650
C -4.5093150 -0.5974110 1.6041350
H -3.1272380 0.5479030 3.7034480
C -4.5316320 -3.3718340 1.3500620
H -2.4684470 -3.2773050 1.9773990
C -5.6752790 -1.2536240 1.2042220
H -4.4979940 0.4855090 1.6886690
C -5.6926680 -2.6438020 1.0744230
H -4.5374150 -4.4562510 1.2655040
H -6.5698060 -0.6751450 0.9857930
H -6.6000590 -3.1550200 0.7630630
O -1.4431820 0.2612530 -0.7983740
C -1.8315630 1.4178740 -1.0451200
C -3.0472970 1.5622220 -1.8981690
C -3.4699360 2.7956940 -2.4248830
C -3.8027780 0.4085680 -2.1804290
C 0.3683970 2.4020010 -0.1402980
C -4.6111690 2.8695410 -3.2214140
H -2.9100020 3.7022520 -2.2243740
C -4.9451560 0.4862700 -2.9699500
H -3.4834770 -0.5376420 -1.7596740
H 0.8199050 2.0194040 -1.0710570
C -5.3504580 1.7168830 -3.4952810
H -4.9225840 3.8273110 -3.6283530
H -5.5228350 -0.4108640 -3.1734370
H -6.2413960 1.7776550 -4.1146020
C -1.1478580 2.6451170 -0.4685110
O 0.5352610 1.4957870 0.9063190
C 1.0464310 3.7394200 0.1582330
C 1.3734640 4.1054820 1.4688250
C 1.3594120 4.6249480 -0.8826140
C 1.9871420 5.3321260 1.7337720
H 1.1462970 3.4100820 2.2698270
C 1.9727620 5.8511760 -0.6222880
H 1.1353640 4.3443600 -1.9107540
C 2.2876950 6.2103910 0.6906650
H 2.2339250 5.6017390 2.7581560
H 2.2141120 6.5209890 -1.4441450
H 2.7691310 7.1630060 0.8967610
H -1.1913090 3.4369540 -1.2255010
O 2.0269590 -1.0404060 1.7855050
C 1.8157080 -1.8371530 2.9889740
C 3.1156180 -0.0761470 2.0003030
C 2.4495300 -1.0205370 4.1086800
H 0.7402140 -1.9891750 3.0795930
H 2.3280100 -2.7980230 2.8508510
C 3.6538200 -0.3777310 3.4011740
H 3.8481570 -0.2265890 1.2060030
H 2.6723640 0.9178900 1.9084420
H 2.7344520 -1.6411850 4.9638140
H 1.7494150 -0.2535690 4.4556990
H 4.4887570 -1.0863490 3.3504590
H 4.0107180 0.5276310 3.9009510
In 0.3340000 -0.4339780 0.4325440
O 0.3277380 -2.6950100 -0.1171090
C 1.4982330 -3.5514640 -0.1992560
C -0.8034280 -3.3104780 -0.7954370
C 1.1460470 -4.6058800 -1.2449080
H 2.3572910 -2.9342810 -0.4624430
H 1.6599810 -3.9977950 0.7898180
C -0.3722080 -4.7522530 -1.0571550
H -1.6734500 -3.2080250 -0.1471970
H -0.9783270 -2.7673850 -1.7288310
H 1.6952960 -5.5401950 -1.0943160
H 1.3713610 -4.2211700 -2.2453040
H -0.5941390 -5.3857780 -0.1899520
H -0.8764270 -5.1798680 -1.9288900
C 0.0827010 -1.2266040 -3.7446810
H -0.3752540 -2.1913110 -4.0126760
H -0.4977010 -0.7839540 -2.9316530
H -0.0385900 -0.5802010 -4.6272920
C -0.9660550 1.0268740 3.9027660
H -0.5544260 0.4694910 4.7602300
H -0.1659870 1.1738450 3.1713150
H -1.2540360 2.0145640 4.2838430
C -1.9566600 3.1160650 0.7632610
H -2.9903920 3.3587860 0.4946290
H -1.9658280 2.3383790 1.5303930
H -1.4880940 4.0132190 1.1784970

TS-A1-A2

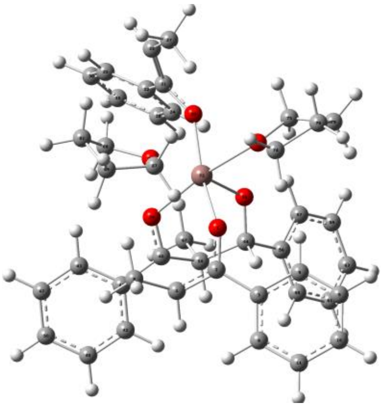

Energy: -2083.22003284 a.u.
Frequency: -252.9078 cm^{-1}
O 0.9865330 -1.1574100 -1.5153630
C 1.9213890 -0.3112690 -1.7971430
C 3.3297350 -0.7651960 -1.5544890
C 1.6327370 0.9637660 -2.3411330
C 3.5828500 -1.7928070 -0.6277590
C 4.4077880 -0.2468360 -2.2904360
H 2.4933780 1.6114900 -2.4895370
C 0.5837190 1.0472690 -3.4333270
C 4.8741970 -2.2709350 -0.4286670
H 2.7540770 -2.1767760 -0.0428970
C 5.7022690 -0.7310990 -2.0957780
H 4.2317970 0.5189660 -3.0390250
H 0.4335400 2.0790950 -3.7598210
H 0.8946170 0.4612090 -4.3106040
H -0.3734390 0.6544760 -3.0857150
C 5.9400790 -1.7426260 -1.1644800
H 5.0548200 -3.0507980 0.3063520
H 6.5232600 -0.3225470 -2.6789440
H 6.9482030 -2.1187560 -1.0120910
O -2.2331590 -1.1951930 0.9603360
C -3.5212470 -0.8903540 1.1480340
C -3.7981700 0.3761730 1.8936340
C -4.5290950 -1.7239470 0.7819720
C -2.8256400 0.8849730 2.7706630
C -5.0101940 1.0778630 1.7693020
H -5.5434230 -1.4624860 1.0698510
C -4.3306790 -3.0398810 0.0877010
C -3.0687120 2.0375810 3.5189560
H -1.8780130 0.3626380 2.8538990
C -5.2519580 2.2306480 2.5149070
H -5.7622240 0.7267500 1.0683550
H -3.2756660 -3.1893280 -0.1577170
H -4.6550860 -3.8842150 0.7152970
H -4.9161480 -3.1105840 -0.8424270
C -4.2838600 2.7146750 3.3987100
H -2.3039920 2.4079670 4.1974080
H -6.1952870 2.7589450 2.3975820
H -4.4712400 3.6150260 3.9782890
O -0.6385340 1.1934340 -0.7603040
C 0.4328270 1.9243530 -0.7151950
C 0.3290000 3.2590860 -1.4171730
C 1.4336390 4.1080010 -1.6001310
C -0.9272510 3.6924570 -1.8671860
C 1.6802410 0.5802380 1.1867500
C 1.2869570 5.3456360 -2.2229760
H 2.4190130 3.7966510 -1.2668390
C -1.0757640 4.9346800 -2.4854560
H -1.7844990 3.0485160 -1.7058960
H 2.4010020 0.1882100 0.4558800
C 0.0302330 5.7655670 -2.6686750
H 2.1552130 5.9850410 -2.3594240
H -2.0594610 5.2556890 -2.8186560
H -0.0838200 6.7331080 -3.1502540
C 1.2388250 1.9642960 0.6037820
O 0.6328620 -0.3147990 1.3925740
C 2.4939240 0.7746870 2.4699130
C 1.9796460 0.3934340 3.7138400
C 3.7839990 1.3213600 2.4178740
C 2.7289910 0.5692650 4.8795230
H 0.9894090 -0.0477730 3.7476180
C 4.5351410 1.5004700 3.5802430
H 4.2121020 1.5981370 1.4558710
C 4.0079870 1.1259640 4.8187630
H 2.3126810 0.2685940 5.8383420
H 5.5350780 1.9235210 3.5184280
H 4.5919530 1.2611700 5.7257590
C 0.3818210 2.7614350 1.6143500
H 0.1234880 3.7522800 1.2289380
H -0.5449620 2.2277780 1.8355410
H 0.9382620 2.8903490 2.5470420
H 2.1594400 2.5291570 0.4206450
In -0.6046670 -0.7937190 -0.0890640
O -0.2417630 -3.0200090 0.2593340
C 0.3404500 -4.0590350 -0.5682160
C -0.2542580 -3.4194890 1.6656430
C 0.5271370 -5.2605660 0.3625090
H -0.3447620 -4.2594630 -1.3966370
H 1.2833470 -3.6838960 -0.9734770
C 0.7147680 -4.5951040 1.7357250
H 0.0528860 -2.5488530 2.2457270
H -1.2793090 -3.6965890 1.9270020
H 1.3750320 -5.8837910 0.0627470
H -0.3714380 -5.8882900 0.3645990
H 1.7435300 -4.2345740 1.8516340
H 0.4924980 -5.2653910 2.5712890
O -1.8904200 -1.2323590 -1.9594170
C -1.5786560 -2.0961920 -3.0826950
C -3.0581680 -0.4032570 -2.2546780
C -2.3855660 -1.5369850 -4.2526310
H -0.4976670 -2.0737350 -3.2235890
H -1.8975280 -3.1151590 -2.8283770
C -3.6329150 -0.9779180 -3.5491570
H -3.7339950 -0.4674840 -1.4003450
H -2.7069150 0.6259070 -2.3743150
H -2.6173250 -2.3033760 -4.9984090
H -1.8307660 -0.7327300 -4.7484760
H -4.3409720 -1.7845620 -3.3274370
H -4.1554270 -0.2188820 -4.1388130

A2

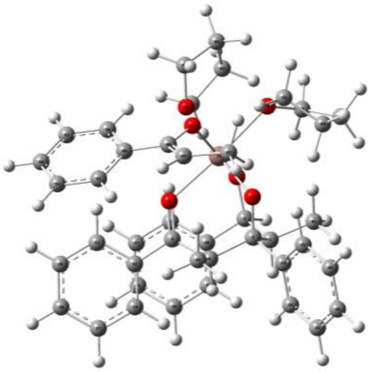

Energy: -2083.23704309 a.u.
C 1.1776820 1.2982070 -2.1080690
C 1.5962540 -0.2140020 -2.2047460
C 1.8029240 0.3491550 1.0828080
C 1.4982020 1.7247010 0.5189570
C 0.5500050 1.8417480 -0.7542860
H 1.7230230 -0.3621620 -3.2946010
H 2.0830080 1.8848170 -2.2991170
H 2.4452530 2.2148740 0.2814630
O 0.9872550 -0.5895050 1.0402420
O 0.6281910 -1.1307420 -1.7826370
O -0.6840850 1.2470110 -0.4996530
C 3.0394090 0.1436880 1.8967810
C 3.0612830 -0.9366290 2.7978710
C 4.1636200 0.9818040 1.8169030
C 4.1708010 -1.1645520 3.6047580
H 2.1912240 -1.5807670 2.8567500
C 5.2796090 0.7441510 2.6179790
H 4.1876250 1.8077890 1.1160080
C 5.2843210 -0.3236880 3.5165850
H 4.1686580 -1.9950980 4.3052830
H 6.1463380 1.3940940 2.5381520
H 6.1525580 -0.5012640 4.1457410
C 0.3543510 3.3722030 -0.9631680
C 1.4308880 4.2362570 -1.2197960
C -0.9293340 3.9246740 -0.9002360
C 1.2303960 5.6054180 -1.4033120
H 2.4444170 3.8462010 -1.2820550
C -1.1357080 5.2936490 -1.0864160
H -1.7603270 3.2572570 -0.7051720
C -0.0570480 6.1424900 -1.3377140
H 2.0821740 6.2522620 -1.5997100
H -2.1441600 5.6975540 -1.0329710
H -0.2153340 7.2082340 -1.4812270
C 3.0075100 -0.5343970 -1.6635420

C 3.2483410 -1.7254530 -0.9683220
C 4.1163900 0.2680920 -1.9849090
C 4.5393480 -2.0948460 -0.5851530
H 2.4014170 -2.3561670 -0.7292100
C 5.4086430 -0.0946220 -1.6027520
H 3.9813690 1.1780000 -2.5644550
C 5.6275160 -1.2797230 -0.8963580
H 4.6938730 -3.0230060 -0.0399190
H 6.2472040 0.5459420 -1.8663340
H 6.6333880 -1.5648360 -0.5991810
O -2.1116340 -1.0497730 1.0139520
C -2.2374430 -0.7914090 2.3292330
C -1.6500190 -1.5116610 3.3147240
H -1.8352200 -1.1995640 4.3391050
C -3.1411470 0.3527380 2.6688720
C -3.1283000 1.5196190 1.8851980
C -4.0242180 0.2958000 3.7599120
C -3.9517900 2.6005160 2.2015630
H -2.4411270 1.5821300 1.0462600
C -4.8525660 1.3745800 4.0716050
H -4.0740660 -0.6124980 4.3538780
C -4.8197860 2.5343710 3.2944230
H -3.9107920 3.5021580 1.5943090
H -5.5332920 1.3039540 4.9169010
H -5.4659000 3.3749120 3.5353990
O -0.9120840 -3.1007530 -0.5152590
C 0.0487270 -4.0051480 -1.1272010
C -2.1393500 -3.8078390 -0.1800910
C -0.7495280 -5.2635510 -1.4649040
H 0.4883370 -3.4809160 -1.9748450
H 0.8334070 -4.2166790 -0.3899540
C -1.8003160 -5.2887510 -0.3437620
H -2.4319560 -3.5081790 0.8246090
H -2.9171120 -3.4835450 -0.8808590
H -0.1205020 -6.1584830 -1.4931200
H -1.2353270 -5.1608440 -2.4432670
H -1.3622320 -5.6852390 0.5797890
H -2.6816210 -5.8904460 -0.5862550
In -0.7318160 -0.7587190 -0.3741760
O -2.4348750 -0.9440630 -1.8557970
C -2.2857650 -1.4714060 -3.2015110
C -3.5690580 -0.0325290 -1.7733530
C -3.5546690 -1.0421410 -3.9404610
H -1.3746430 -1.0497980 -3.6322020
H -2.1667420 -2.5552350 -3.1224200
C -3.9332180 0.2664580 -3.2258980
H -4.3723670 -0.5426950 -1.2324940
H -3.2473620 0.8392410 -1.2036430
H -4.3468740 -1.7893240 -3.8128380
H -3.3809740 -0.9127870 -5.0127930
H -4.9886810 0.5295220 -3.3426170
H -3.3289130 1.0980210 -3.6052000
C -0.7536740 -2.7002130 3.1249500
H -1.2170090 -3.6271600 3.4996530
H -0.5072480 -2.8518160 2.0709980
H 0.1899470 -2.5880240 3.6786530
C 0.2023310 1.5965580 -3.2654750
H -0.0092580 2.6645490 -3.3561550
H 0.6252570 1.2531490 -4.2169480
H -0.7489810 1.0834850 -3.1044800
C 0.8761410 2.4707700 1.7395180
H 1.4982950 2.3593800 2.6332240
H 0.7863000 3.5355590 1.5169630
H -0.1187420 2.0727450 1.9515380

TS-A2-A3

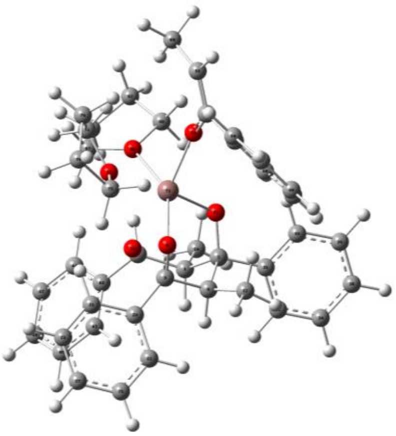

Energy: -2083.22454263 a.u.
Frequency: -142.2961 cm^{-1}
C 2.4588870 1.0994500 -1.0332300
C 2.2650980 -0.4309970 -0.8444380
C 0.9059080 0.3135310 1.5813110
C 1.2507410 1.7332560 1.0949370
C 1.2070930 1.8729200 -0.4841890
H 1.8250090 -0.7923490 -1.7806390
H 3.3299600 1.4158190 -0.4457120
H 2.2827640 1.9517500 1.3864870
O -0.3726860 0.0212150 1.6386240
O 1.3041830 -0.7614740 0.1665530
O 0.0215640 1.3777950 -1.0425670
C 0.3194910 2.7187130 1.8266510
H 0.3045770 2.5229950 2.9044740
H 0.6503180 3.7479750 1.6677890
H -0.7042810 2.6174870 1.4638170
C 2.7611770 1.3875320 -2.5100820
H 3.1014210 2.4155590 -2.6583670
H 3.5443360 0.7164580 -2.8833290
H 1.8610390 1.2423290 -3.1186470
C 1.7171420 -0.2864600 2.7110160
C 1.6231800 -1.6667860 2.9501830
C 2.4952660 0.4927620 3.5757880
C 2.3007060 -2.2525010 4.0149830
H 1.0399830 -2.2723260 2.2661210
C 3.1672550 -0.0936770 4.6538270
H 2.5733470 1.5649960 3.4292670
C 3.0751720 -1.4661820 4.8750410
H 2.2312320 -3.3255290 4.1746730
H 3.7603100 0.5290010 5.3185120
H 3.6022550 -1.9230650 5.7085150
C 1.3556110 3.3666640 -0.8441970
C 2.4638850 4.1263370 -0.4335480
C 0.3736820 3.9981770 -1.6150440
C 2.5816080 5.4734850 -0.7774200
H 3.2495350 3.6690950 0.1635190
C 0.4884800 5.3470630 -1.9615030
H -0.4763050 3.4083520 -1.9373210
C 1.5914350 6.0923770 -1.5446080
H 3.4487290 6.0396870 -0.4456010
H -0.2895160 5.8156120 -2.5600070
H 1.6815060 7.1419100 -1.8131480
C 3.5784850 -1.1815770 -0.6603980
C 4.3516680 -1.0502760 0.5028370
C 4.0562920 -2.0179580 -1.6777500
C 5.5571360 -1.7367670 0.6419670
H 4.0046860 -0.4156490 1.3112400
C 5.2674700 -2.7030100 -1.5473910
H 3.4763410 -2.1313710 -2.5915910
C 6.0219410 -2.5657310 -0.3830970
H 6.1359730 -1.6239440 1.5550900
H 5.6168600 -3.3428140 -2.3540080
H 6.9631850 -3.0980030 -0.2733640
O -2.6453480 -0.1100320 -1.1117300
C -3.8887730 0.0002910 -0.6153600
C -4.9491790 -0.6485230 -1.1592870
H -5.9354390 -0.4198460 -0.7650230
C -4.0924200 0.9654650 0.5072490
C -3.3331440 2.1471090 0.5541760
C -5.0493510 0.7484000 1.5143720
C -3.5460610 3.0923900 1.5582670
H -2.5808890 2.3216900 -0.2096430
C -5.2564030 1.6906890 2.5214390
H -5.6266190 -0.1722170 1.5085390
C -4.5087220 2.8703650 2.5451680
H -2.9576560 4.0062480 1.5675790
H -5.9976520 1.4995680 3.2937300
H -4.6692630 3.6050710 3.3298660
C -4.8894080 -1.5663520 -2.3455440
H -3.8588620 -1.6986240 -2.6879500
H -5.3074260 -2.5608190 -2.1256290
H -5.4703670 -1.1705840 -3.1918470
In -0.8128700 -0.3059890 -0.3521310
O -1.6088610 -2.3014090 0.5978350
C -2.3434330 -3.3048520 -0.1560110
C -2.1163740 -2.2475160 1.9752090
C -3.5435190 -3.6780750 0.7104430
H -2.6145640 -2.8689820 -1.1172200
H -1.6754630 -4.1620750 -0.3100310
C -2.9794230 -3.4966470 2.1267870
H -1.2573540 -2.1974280 2.6412310
H -2.7017780 -1.3305880 2.0872830
H -3.9006670 -4.6926500 0.5091060
H -4.3615930 -2.9731800 0.5287960
H -2.3669840 -4.3604970 2.4125630
H -3.7551780 -3.3639200 2.8867940
O -0.4864510 -1.5398190 -2.2292150
C 0.1925450 -2.7981310 -2.4868200
C -0.8170090 -0.8603450 -3.4883020
C 0.5904820 -2.7343440 -3.9600560
H 1.0297740 -2.8817320 -1.7909440

H -0.5141460 -3.6137120 -2.2984680
C -0.5491910 -1.9021480 -4.5711490
H -1.8511440 -0.5276700 -3.4033910
H -0.1622080 0.0118860 -3.5677150
H 0.6878470 -3.7296570 -4.4034030
H 1.5469250 -2.2122520 -4.0770680
H -1.4350580 -2.5269180 -4.7347750
H -0.2786370 -1.4405270 -5.5250660

A3

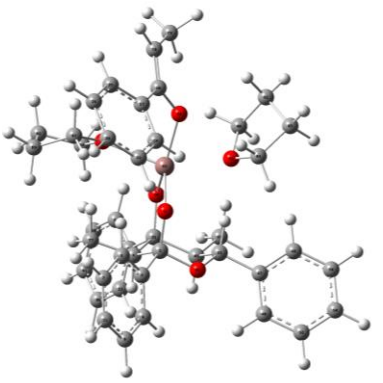

Energy: -2083.24037002 a.u.
O 0.7201040 -0.9946040 -1.5881570
C 1.6280610 -0.0825640 -1.1469660
C 3.0511220 -0.4330340 -1.5942720
C 1.2260360 1.3850190 -1.4938010
C 3.2477880 -1.5151210 -2.4605360
C 4.1681360 0.3203400 -1.2059380
C 4.5258940 -1.8501160 -2.9130820
H 2.3799320 -2.0780140 -2.7830260
C 5.4466570 -0.0147050 -1.6515620
H 4.0520360 1.1656190 -0.5357620
C 5.6318970 -1.1027430 -2.5076440
H 4.6549670 -2.6923550 -3.5888250
H 6.2990540 0.5768690 -1.3278120
H 6.6280040 -1.3601250 -2.8586670
O -1.5135930 -1.3390790 1.6251560
C -2.2304360 -0.5519020 2.4644730
C -3.5895210 -0.1460100 1.9980620
C -1.8090840 -0.2437570 3.7115930
C -4.3769480 -1.0447900 1.2583790
C -4.1331350 1.1130830 2.3043410
H -2.4906860 0.3134270 4.3488880
C -5.6720950 -0.7070650 0.8623390
H -3.9699210 -2.0213050 1.0147290
C -5.4242720 1.4547170 1.9020370
H -3.5266920 1.8325020 2.8464050
C -6.2026780 0.5454880 1.1815310
H -6.2724470 -1.4276460 0.3111600
H -5.8201440 2.4378510 2.1444300
H -7.2095930 0.8110080 0.8699540
O -0.8933200 1.1208920 -0.2415680
C 0.2142780 1.9592730 -0.4351980
C -0.2767140 3.3555160 -0.8759330
C 0.6133070 4.3777470 -1.2449530
C -1.6466220 3.6434660 -0.8863390
C 1.6294750 0.7871790 1.3655660
C 0.1488320 5.6380990 -1.6236000
H 1.6850950 4.1948920 -1.2468520
C -2.1155120 4.9048370 -1.2622610
H -2.3341990 2.8651990 -0.5770640
H 0.9757620 0.4165840 2.1631610
C -1.2214260 5.9083370 -1.6359890
H 0.8599630 6.4094140 -1.9097390
H -3.1850530 5.1028190 -1.2593520
H -1.5847480 6.8896630 -1.9305180
C 1.0371800 2.1389380 0.8903170
O 1.5070140 -0.2801190 0.3820140
C 3.0377640 0.8298730 1.9613140
C 3.6358840 2.0137530 2.4132690
C 3.7356950 -0.3748110 2.1429230
C 4.8974790 1.9964890 3.0167140
H 3.1229140 2.9637600 2.3060640
C 4.9938120 -0.3942250 2.7406700
H 3.2888820 -1.2973740 1.7880720
C 5.5827460 0.7941630 3.1812590
H 5.3405490 2.9291340 3.3566370
H 5.5180030 -1.3390180 2.8608270
H 6.5641320 0.7807200 3.6480950
H 1.8613700 2.8177620 0.6426600
O -2.3017100 -1.3001520 -1.5807170
C -3.1491440 -0.1846970 -2.0356340
C -2.0092240 -2.1992200 -2.6954120
C -3.2018380 -0.3218410 -3.5556080
H -4.1263660 -0.3158540 -1.5642650
H -2.6803920 0.7327500 -1.6781620
C -3.0316770 -1.8346620 -3.7672140
H -0.9809860 -2.0093750 -3.0161950
H -2.0955620 -3.2209220 -2.3202480
H -2.3721950 0.2233440 -4.0189430
H -4.1376070 0.0667670 -3.9680170
H -2.6809160 -2.0925010 -4.7709880
H -3.9788740 -2.3576080 -3.5904430
H 2.1299070 2.0018430 -1.4391900
O -0.3743400 -3.1879010 -0.1210130
C 0.8906340 -3.7792180 -0.5529210
C -1.0871860 -4.1165920 0.7449070
C 0.9247780 -5.1810390 0.0664640
H 0.9123220 -3.7801510 -1.6441860
H 1.6964940 -3.1360530 -0.1953030
C -0.0054010 -5.0456380 1.2817470
H -1.5947090 -3.5130980 1.4947030
H -1.8204450 -4.6638930 0.1361430
H 1.9423530 -5.4835330 0.3308350
H 0.5252250 -5.9240220 -0.6340360
H 0.5161910 -4.5690110 2.1196450
H -0.4081730 -6.0026290 1.6270300
C -0.5050210 -0.6741090 4.3159210
H -0.6639780 -1.2304860 5.2508500
H 0.0499160 -1.3218460 3.6314260
H 0.1355970 0.1827790 4.5720820
C 0.7194530 1.4320730 -2.9416980
H -0.2466450 0.9292090 -3.0265280
H 0.6044390 2.4655890 -3.2785410
H 1.4208830 0.9267270 -3.6143510
C 0.2131470 2.7620560 2.0262260
H -0.6832820 2.1612970 2.2104570
H 0.7918130 2.8022510 2.9571170
H -0.0980130 3.7808060 1.7777100
In -0.6779620 -0.8667670 -0.1122190

A2$_{OH}$

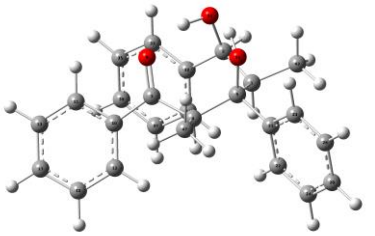

Energy: -1193.9686888 a.u.
C 1.3282800 -1.6016070 0.2762880
C 0.0688490 -2.4183390 -0.1634580
C -0.7819620 0.6604100 -1.0741520
C 0.6106420 0.8812000 -0.4895690
C 1.6778540 -0.2904790 -0.5334280
H 0.2702750 -3.4444820 0.1700130
H 1.1897480 -1.3001470 1.3188440
H 0.4975080 1.1629590 0.5606730
O -0.9829030 -0.0793400 -2.0373980
C -1.9292470 1.4810500 -0.5525640
C -3.1764940 1.3145530 -1.1786580
C -1.8225420 2.4030040 0.5023110
C -4.2846180 2.0458740 -0.7639560
H -3.2498180 0.6022580 -1.9925280
C -2.9327900 3.1373540 0.9161870
H -0.8747970 2.5648550 1.0042140
C -4.1654690 2.9608050 0.2853590
H -5.2418930 1.9050630 -1.2583730
H -2.8339340 3.8489970 1.7311840
H -5.0295810 3.5346570 0.6096990
C 2.9590780 0.3056060 0.0966670
C 2.9808910 0.7372520 1.4317070
C 4.1311600 0.4301570 -0.6587070
C 4.1372950 1.2773280 1.9950800
H 2.0912050 0.6490880 2.0510270
C 5.2890100 0.9727280 -0.0970500
H 4.1238130 0.0933190 -1.6883080
C 5.2990440 1.3993030 1.2309800
H 4.1291520 1.6008060 3.0329230
H 6.1873070 1.0597100 -0.7032550
H 6.2009750 1.8199320 1.6676250
C -1.2829730 -2.0566450 0.4642180
C -2.4620140 -2.4072900 -0.2122010
C -1.4019060 -1.4998500 1.7450830
C -3.7133970 -2.1869640 0.3606580
H -2.3892710 -2.8592850 -1.1957890
C -2.6552600 -1.2796480 2.3219510
H -0.5173320 -1.2409540 2.3186120
C -3.8173780 -1.6179960 1.6315480
H -4.6102140 -2.4660000 -0.1867370
H -2.7176970 -0.8418880 3.3149520
H -4.7924070 -1.4448830 2.0788960

C 2.5269410 -2.5766150 0.2381820
H 3.4589330 -2.0997730 0.5462650
H 2.3404440 -3.4197830 0.9129770
H 2.6688230 -2.9773660 -0.7700460
C 1.1449370 2.1137360 -1.2745380
H 0.4008100 2.9153200 -1.3102960
H 2.0489900 2.5081800 -0.8060450
H 1.3827400 1.8111150 -2.2973420
O 1.9870660 -0.6234940 -1.8794770
H 1.4176740 -1.3796840 -2.1163560
O -0.0096520 -2.5431680 -1.5903640
H -0.4751160 -1.7435600 -1.9225300

TS-A2$_{OH}$-5a

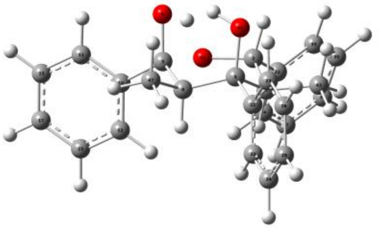

Energy: -1193.9230456 a.u.
Frequency: -1659.3900 cm^{-1}
C -0.6680690 1.2396410 -0.0075200
C 0.6744500 1.3544120 -0.7546650
C 0.6160780 -1.4156390 -0.7913350
C -0.7628860 -1.3185030 -0.1465510
C -1.4928590 0.0065390 -0.5257940
H 0.4374210 1.5771020 -1.8012670
H -0.4553750 1.0858900 1.0597150
H -0.6392800 -1.3272170 0.9407860
O 0.6551920 -1.4080410 -2.1381710
C 1.6149260 -2.3198920 -0.1080560
C 2.2970030 -3.2652760 -0.8815960
C 1.8679220 -2.2503910 1.2693440
C 3.2105740 -4.1364360 -0.2863790
H 2.0932600 -3.3044880 -1.9460650
C 2.7865560 -3.1138210 1.8613720
H 1.3626920 -1.5067440 1.8797230
C 3.4576810 -4.0626810 1.0845870
H 3.7298930 -4.8715520 -0.8953340
H 2.9819130 -3.0459720 2.9282100
H 4.1710600 -4.7386740 1.5484730
C -2.8900910 0.0101290 0.1089950
C -3.0509280 -0.0403880 1.5024080
C -4.0360690 0.0596760 -0.6922510
C -4.3213170 -0.0426950 2.0781250
H -2.1806050 -0.0764440 2.1534110
C -5.3086810 0.0597680 -0.1164540
H -3.9166850 0.0960880 -1.7684990
C -5.4583460 0.0078710 1.2691400
H -4.4214620 -0.0835380 3.1597880
H -6.1859570 0.0992570 -0.7573890
H -6.4489990 0.0066410 1.7160030
C 1.6049700 2.4242700 -0.2190590
C 1.8099380 3.6078370 -0.9365650
C 2.2741680 2.2487290 0.9996130
C 2.6498340 4.6070760 -0.4402230
H 1.3078090 3.7495740 -1.8909200
C 3.1165790 3.2421900 1.4951560
H 2.1513910 1.3189370 1.5479120
C 3.3041570 4.4269800 0.7782800
H 2.7969590 5.5205990 -1.0101870
H 3.6324800 3.0904500 2.4396710
H 3.9625950 5.2003640 1.1646010
C -1.4495140 2.5529180 -0.1750460
H -2.3542520 2.5490300 0.4367920
H -0.8440650 3.4140190 0.1211480
H -1.7511460 2.6823500 -1.2201830
C -1.5496960 -2.5812180 -0.5535950
H -0.9869540 -3.4848910 -0.2989970
H -2.5082060 -2.6130270 -0.0304650
H -1.7403420 -2.5873320 -1.6295830
O -1.6356290 0.0968810 -1.9407460
H -0.9037020 -0.4330620 -2.3312900
O 1.4226670 0.0939430 -0.7398450
H 1.3934260 -0.3861750 -1.8375360

5a

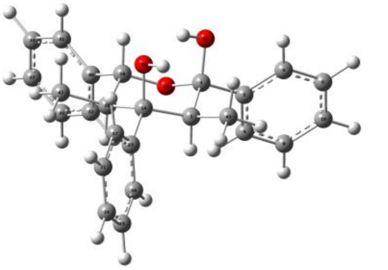

Energy: -1193.985187 a.u.
C -0.9247130 -1.0862990 -0.6698410
C -1.9031190 -2.1143980 -0.1088500
C 0.5273870 -1.3174590 -0.1399680
C -2.3855040 -3.1578150 -0.9052440
C -2.3108170 -2.0357920 1.2289340
C -3.2577690 -4.1087200 -0.3711310
H -2.0825150 -3.2109260 -1.9445630
C -3.1798270 -2.9867260 1.7625100
H -1.9610570 -1.2140280 1.8455960
C -3.6554070 -4.0288120 0.9635690
H -3.6288360 -4.9117340 -1.0029990
H -3.4911720 -2.9098330 2.8011770
H -4.3341960 -4.7696820 1.3781590
C 1.4612330 -0.1308440 -0.5434020
C 2.8451600 -0.2548760 0.1119100
C 2.9673540 -0.4510080 1.4968570
C 4.0206540 -0.1475680 -0.6432890
C -0.6532770 1.3113140 -0.6497950
C 4.2196090 -0.5524390 2.1009860
H 2.0788490 -0.5247660 2.1175520
C 5.2765870 -0.2525900 -0.0406270
H 3.9587150 0.0465830 -1.7091400
H -0.6678850 1.3248370 -1.7477530
C 5.3823740 -0.4590290 1.3335000
H 4.2855690 -0.7060880 3.1748460
H 6.1717870 -0.1650900 -0.6507530
H 6.3582860 -0.5407150 1.8040340
C 0.8168800 1.2390310 -0.1693340
O -1.3883370 0.1772420 -0.1832960
C -1.3798310 2.5446290 -0.1480980
C -1.6563600 3.6095790 -1.0118900
C -1.7769500 2.6431230 1.1920080
C -2.3036970 4.7566100 -0.5479400
H -1.3671420 3.5387710 -2.0579390
C -2.4280930 3.7847360 1.6573120
H -1.5913030 1.8108950 1.8644030
C -2.6905530 4.8474740 0.7892480
H -2.5120500 5.5736110 -1.2338340
H -2.7356200 3.8439770 2.6982730
H -3.1993220 5.7366380 1.1522280
H 0.7888380 1.2681130 0.9278950
H 0.4451140 -1.3094930 0.9516690
C 1.0886820 -2.6817640 -0.5679790
H 1.0906740 -2.7946630 -1.6582080
H 2.1084010 -2.8259020 -0.1976160
H 0.4673880 -3.4890940 -0.1718190
C 1.6406330 2.4319740 -0.6724980
H 1.6822640 2.4472620 -1.7661610
H 1.1965500 3.3715180 -0.3328250
H 2.6672640 2.3878170 -0.2983100
O -0.9698280 -1.1477060 -2.0748240
H -0.1483660 -0.7296400 -2.3999910
O 1.5926910 -0.1230430 -1.9904050
H 2.0997470 -0.9143060 -2.2374070

6a

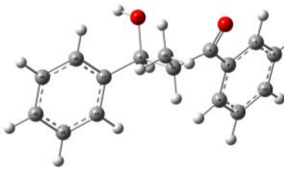

Energy: -769.7775468 a.u.
O 1.7472810 -2.4195030 -0.4116820
C 1.3885250 -1.2541130 -0.4507820
C 2.3910020 -0.1578400 -0.2041200
C 2.1816850 1.1842260 -0.5576010
C 3.6141340 -0.5192370 0.3842560
C -0.7280300 -0.2984080 0.5409350
C 3.1701290 2.1414570 -0.3251940
H 1.2560610 1.4898800 -1.0353400
C 4.5958140 0.4365520 0.6260680
H 3.7690030 -1.5619030 0.6412890
H -0.1072460 0.5548070 0.8542750
C 4.3760250 1.7709560 0.2712910
H 2.9987260 3.1752310 -0.6128880
H 5.5347770 0.1440050 1.0883570
H 5.1436740 2.5178560 0.4558390
C -0.0791260 -0.8967630 -0.7424640
C -2.1421300 0.2193760 0.3131950
C -3.2606070 -0.5719350 0.6014200
C -2.3426260 1.5123350 -0.1880470
C -4.5505210 -0.0834050 0.3846550
H -3.1103210 -1.5708200 0.9990970
C -3.6297760 2.0006390 -0.4106810

H -1.4833320 2.1472600 -0.3971490
C -4.7395500 1.2021920 -0.1243060
H -5.4087890 -0.7097220 0.6140820
H -3.7669890 3.0069830 -0.7977780
H -5.7436890 1.5824580 -0.2916070
H -0.0971500 -0.0947610 -1.4924390
O -0.6708770 -1.3196150 1.5322330
H -1.0450810 -0.9554540 2.3500760
C -0.8305480 -2.1123400 -1.2922140
H -0.8447780 -2.9192260 -0.5571000
H -1.8591140 -1.8455440 -1.5512170
H -0.3333880 -2.4902620 -2.1903670

6ac

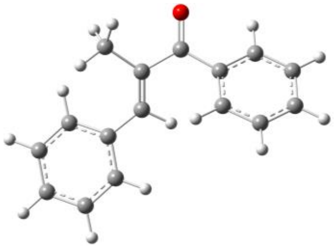

Energy: -693.3482976 a.u.
O 1.8405640 2.5545940 -0.1996940
C 1.3798680 1.4341440 0.0021230
C 2.3054030 0.2486560 0.0004410
C 2.1299270 -0.8599410 0.8428350
C 3.4494950 0.3135520 -0.8103820
C -0.7166050 0.1456470 -0.1366110
C 3.0719590 -1.8893960 0.8600120
H 1.2698380 -0.9043120 1.5034820
C 4.3779530 -0.7233960 -0.8077750
H 3.5929470 1.1903030 -1.4337950
H -0.0776890 -0.6612280 -0.4883290
C 4.1910430 -1.8282560 0.0284250
H 2.9331800 -2.7365850 1.5263440
H 5.2523200 -0.6691400 -1.4507990
H 4.9200950 -2.6342590 0.0367680
C -0.0927280 1.2887610 0.2390410
C -2.1435280 -0.2031320 -0.1453920
C -3.1959820 0.7299170 -0.2252450
C -2.4808110 -1.5716860 -0.1060180
C -4.5245380 0.3084250 -0.2341040
H -2.9779080 1.7868850 -0.3153270
C -3.8075920 -1.9913830 -0.1031350
H -1.6828910 -2.3096950 -0.0679140
C -4.8378600 -1.0501360 -0.1635510
H -5.3190330 1.0466290 -0.3041330
H -4.0385070 -3.0524370 -0.0610160
H -5.8752020 -1.3735060 -0.1681960
C -0.7625440 2.5308150 0.7748400
H -1.0840210 3.1988880 -0.0345310
H -1.6398360 2.2823460 1.3785800
H -0.0585560 3.1038130 1.3834020
